# Predictors of Seizure Outcomes in Children with Tuberous Sclerosis Complex and Intractable Epilepsy Undergoing Resective Epilepsy Surgery: An Individual Participant Data Meta-Analysis

**DOI:** 10.1371/journal.pone.0053565

**Published:** 2013-02-06

**Authors:** Aria Fallah, Gordon H. Guyatt, O. Carter Snead, Shanil Ebrahim, George M. Ibrahim, Alireza Mansouri, Deven Reddy, Stephen D. Walter, Abhaya V. Kulkarni, Mohit Bhandari, Laura Banfield, Neera Bhatnagar, Shuli Liang, Federica Teutonico, Jianxiang Liao, James T. Rutka

**Affiliations:** 1 Division of Neurosurgery, Department of Surgery, University of Toronto, Toronto, Canada; 2 Department of Clinical Epidemiology and Biostatistics, McMaster University, Hamilton, Canada; 3 Department of Medicine, McMaster University, Hamilton, Canada; 4 Division of Neurology, Department of Medicine, Hospital for Sick Children, Toronto, Canada; 5 Hospital for Sick Children, Research Institute, Toronto, Canada; 6 Institute of Medical Sciences, University of Toronto, Toronto, Canada; 7 Division of Neurosurgery, Department of Surgery, McMaster University, Hamilton, Canada; 8 Division of Orthopedic Surgery, Department of Surgery, McMaster University, Hamilton, Canada; 9 Health Sciences Library, McMaster University, Hamilton, Canada; 10 Department of Neurosurgery, First Affiliated Hospital of PLA General Hospital, Beijing, China; 11 Department of Child Neurology and Psychiatry, University of Pavia, Pavia, Italy; 12 Department of Pediatric Neurology, Shenzhen Children's Hospital, Shenzhen, China; University of Utah School of Medicine, United States of America

## Abstract

**Objective:**

To perform a systematic review and individual participant data meta-analysis to identify preoperative factors associated with a good seizure outcome in children with Tuberous Sclerosis Complex undergoing resective epilepsy surgery.

**Data Sources:**

Electronic databases (MEDLINE, EMBASE, CINAHL and Web of Science), archives of major epilepsy and neurosurgery meetings, and bibliographies of relevant articles, with no language or date restrictions.

**Study Selection:**

We included case-control or cohort studies of consecutive participants undergoing resective epilepsy surgery that reported seizure outcomes. We performed title and abstract and full text screening independently and in duplicate. We resolved disagreements through discussion.

**Data Extraction:**

One author performed data extraction which was verified by a second author using predefined data fields including study quality assessment using a risk of bias instrument we developed. We recorded all preoperative factors that may plausibly predict seizure outcomes.

**Data Synthesis:**

To identify predictors of a good seizure outcome (i.e. Engel Class I or II) we used logistic regression adjusting for length of follow-up for each preoperative variable.

**Results:**

Of 9863 citations, 20 articles reporting on 181 participants were eligible. Good seizure outcomes were observed in 126 (69%) participants (Engel Class I: 102(56%); Engel class II: 24(13%)). In univariable analyses, absence of generalized seizure semiology (OR = 3.1, 95%CI = 1.2–8.2, p = 0.022), no or mild developmental delay (OR = 7.3, 95%CI = 2.1–24.7, p = 0.001), unifocal ictal scalp electroencephalographic (EEG) abnormality (OR = 3.2, 95%CI = 1.4–7.6, p = 0.008) and EEG/Magnetic resonance imaging concordance (OR = 4.9, 95%CI = 1.8–13.5, p = 0.002) were associated with a good postoperative seizure outcome.

**Conclusions:**

Small retrospective cohort studies are inherently prone to bias, some of which are overcome using individual participant data. The best available evidence suggests four preoperative factors predictive of good seizure outcomes following resective epilepsy surgery. Large long-term prospective multicenter observational studies are required to further evaluate the risk factors identified in this review.

## Introduction

### Problem definition

Tuberous sclerosis complex (TSC) is a genetic, variably expressed and multisystem disorder with a prevalence of 1 in 10,000 [1]. TSC is one of the leading causes of genetic epilepsy with seizures affecting almost 90% of affected individuals [2]. Only a third of these patients will achieve seizure freedom on antiepileptic drugs [3]. If an epileptogenic zone (EZ) associated with one or more tubers, ideally in non-eloquent cortex, can be localized, resective surgery may be offered as a cure. With resective surgery, 57% of children achieve seizure freedom and another 18% experience a reduction (>90%) in seizure frequency at 1-year follow-up [4]. Other benefits include the possibility of decreasing or discontinuing antiepileptic drugs, ability to obtain/retain employment, ability to drive, improved independent functioning and improved social relationships with family and friends.

Resective surgery, however, still leaves a large proportion of children (>40%), who have incurred the risks of brain surgery, with ongoing seizures. Approximately 3% of patients suffer major surgical morbidity [5], [6]. In addition, mortality, including early postoperative death (secondary to hemorrhage, infection and hydrocephalus) and late postoperative death (unexplained or related to seizures) is between 1 to 2% [5], [7]–[9]. Patients with TSC often undergo invasive electroencephalography (EEG) evaluation to accurately localize the EZ and eloquent cortex prior to determination of resective surgery candidacy. This procedure adds additional risks such as neurological deficits, intracranial hypertension and death [10].

Epilepsy surgery outcome studies in children with TSC are associated with methodological challenges, including: 1) Heterogeneous participant cohorts (e.g. demographics and pathology); 2) Predominance of retrospective study designs; and 3) Seizure outcomes commonly reported at point intervals and not adjusted for variable follow-up lengths. Given that seizure recurrence is time dependent, it is statistically more powerful to investigate outcomes using time-to-event (TTE) analysis. In the absence of TTE data, the variable length of follow-up should be adjusted for using multivariate regression models. Given the lack of strong evidence to predict seizure outcomes, clinical decision making regarding selection of surgical candidates and patient/family counseling regarding the risks and benefits of surgery is challenging and variable across centers.

### Literature review

Previous retrospective cohort studies that attempted to identify factors predictive of seizure outcome in children with Tuberous Sclerosis Complex have several limitations: Inclusion of participants who have undergone palliative epilepsy surgery [11], inclusion of participants with variable follow-up lengths [11] and arbitrarily chosen dichotomization of continuous predictor variables [12].

A meta-analysis identified febrile seizures and EEG/MRI concordance as predictors of positive seizure outcomes, and the utilization of invasive EEG as a predictor of negative seizure outcomes [13]. Although the methodological design of this review was robust, it included participants with all epilepsy syndromes, had a predominantly adult population and a large representation of mesial temporal sclerosis, a distinct epilepsy syndrome with favourable surgical outcomes. Therefore, there is minimal transferability of this knowledge to patients with TSC.

A 2007 systematic review of predictors of seizure outcomes following epilepsy surgery for TSC identified the presence of tonic seizures, moderate or severe intellectual disability (IQ<70) and multifocal single-photon emission computed tomography (SPECT) findings as significant predictors of seizure recurrence [4]. This review had several important limitations: studies were not evaluated for risk of bias, seizure outcomes were pooled and analyzed by the last reported outcome (i.e. not adjusted for the variable follow-up length), Chi square assumption was violated (SPECT was analyzed with only 2 patients in one arm), participants with less than 1 year follow-up (i.e. inadequate follow-up) were included in the analysis, and participants who had undergone palliative surgical procedures were included in the analysis (the goal of palliative epilepsy surgery is not seizure freedom).

### Research question

We performed a systematic review and an individual participant data (IPD) meta-analysis addressing the following study question and reported our findings in concordance with the MOOSE guidelines [14]:

‘*In children with Tuberous Sclerosis Complex and intractable epilepsy undergoing resective epilepsy surgery, what preoperative factors are predictive of good seizure outcomes?*’

A decision was made to limit the study population to those undergoing surgery for several reasons: 1) Ensure homogeneity of the patient population; 2) Ensure comparability of seizure outcome after a similar follow-up (i.e. the duration following surgery is better defined compared to the duration following medical intractability; and 3) The most informative study would be a network meta-analysis comparing the efficacy of no therapy, medical therapy, ketogenic diet, palliative surgery and resective surgery. Given the generally low level of evidence, this comparison would be extremely difficult and likely result in low quality data that will not inform decision making.

### Type of Study Design Used

IPD meta-analysis is recognized to be the gold standard methodology for conducting meta-analysis. It will allow us to: 1) Address questions not addressed in the original publications (e.g. determining predictors of outcomes in a study that had an alternative objective); 2) Use common definitions, coding and cutpoints; 3) Ensuring accuracy of aggregate study data; 4) Account for the variability in clinical follow-up times; and 5) Enhance statistical power in identifying participant covariates that predict seizure outcomes.

## Methods

### Protocol and registration

We developed a protocol prior to conduct of the review but did not register it.

### Search Strategy

We used multiple strategies to identify potentially eligible studies: 1) We conducted electronic literature searches of MEDLINE, EMBASE, CINAHL and Web of Science (Appendix S1) for relevant articles from inception to October 2011. We used the following search terms: “tuberous sclerosis”, “epilepsy surgery”, “seizure outcomes”, “Engel classification”, and “predictors”. The search was restricted to humans but with no language limitations (Non-English articles were translated); 2) one reviewer (A.F.) hand searched all abstracts of the American Epilepsy Society, American Neurological Association, American Association of Neurological Surgeons, Congress of Neurological Surgeons, Canadian Neurological Sciences Federation and European Association of Neurosurgical Societies meetings from 2000 to 2011 for any relevant unpublished literature; 3) one reviewer (A.F.) manually searched the bibliography of our included studies and used the “related articles” feature of PubMed; and 4) we consulted content experts (J.T.R. and O.C.S.) for additional relevant articles.

### Eligibility criteria

Inclusion criteria for the studies were the following:

- [1] Case-control or cohort methodology.
- [2] Consecutive participants.
- [3] At least 90% of participants are less than 19 years of age at the time of surgery.
- [4] At least 90% of participants have TSC.
- [5] At least 90% of participants have undergone resective epilepsy surgery.
- [6] Seizure outcomes reported.
- [7] When etiology is not reported in the title or abstract for a pediatric cohort of greater than 10 participants undergoing resective epilepsy surgery, a full-text review of these articles was performed to determine if they met eligibility criteria.

Exclusion criteria for the studies were the following:

- [1] Single case reports.
- [2] Reviews.
- [3] Mixed adult and pediatric epilepsy surgery studies that do not mention TSC in the title and abstract.
- [4] Participants with anomalous features.
- [5] Participants that have undergone previous epilepsy surgery.
- [6] Participants that have undergone resective epilepsy surgery while in status epilepticus or epilepsia partialis continua.
- [7] Participants with normal MRI.
- [8] Participants that have undergone palliative surgical procedures (i.e. corpus collosotomy, multiple subpial transection or vagal nerve stimulator insertion).

Two teams of 2 reviewers (S.E., A.F., G.M.I., A.M., and D.R.) with methodological and/or content expertise performed title and abstract screening and full text review independently and in duplicate. Reviewers used pilot-tested screening forms and performed pilot calibration exercises to optimize accuracy of eligibility judgments. Reviewers maintained a list of all citations that were excluded after full text review including justifications. Reviewers resolved disagreements through discussion.

### Primary outcome

Our primary outcome was seizure status following resective epilepsy surgery, measured by the Engel Classification scale. We dichotomized the outcome into ‘Good seizure outcome’ (i.e. Engel Class I or II) and ‘Poor seizure outcome’ (i.e. Engel Class III or IV) at the longest reported follow-up time. We adjusted for the variable length of follow-up using a multivariate regression model.

### Selection and coding of data

We recorded all preoperative factors reported in the articles that may plausibly predict seizure outcomes at an individual participant level. A list of biologically plausible predictors was developed *a priori* on the basis of prior literature in consultation with content experts (J.T.R. and O.C.S.) (Table S1). We recorded continuous data where appropriate.

### Data classification and coding

One reviewer (A.F.) performed data abstraction which a second reviewer (A.M.) verified. We contacted corresponding authors of the studies for missing data. Data received from corresponding authors was checked for missing or duplicate data. Participants with missing outcome data (i.e. length of follow-up or Engel classification) were excluded.

### Assessment of study quality

Two reviewers (A.F. and G.M.I.) independently and in duplicate evaluated the risk of bias of each included study (Appendix S2). We evaluated 5 criteria (Sample representativeness, Prognostic variables being well-defined, confidence in outcome assessment, adequacy of follow-up and standardization of treatment) with response options as “definitely yes”, “probably yes”, “probably no” and “definitely no”. ‘Definitely yes’ and ‘probably yes’ responses were assigned a ‘low risk of bias’ while ‘definitely no’ and ‘probably no’ were assigned a ‘high risk of bias’. Judgments were made using a guide we developed apriori. Reviewers resolved all disagreements through discussion.

### Assessment of publication bias and heterogeneity

Data permitting we intended to assess publication bias through visual assessment for symmetry in a funnel plot and a funnel plot regression using the treatment effect as the dependent variable and the reciprocal of the pooled variance for each study as the independent variable [15]. Data permitting, we intended to assess heterogeneity using *I^2^* and Chi square statistics.

### Statistical methods

We calculated Cohen's Kappa score to determine the strength of agreement for full-text review using a computer software (Measurement of clinical agreement for categorical data: The Kappa Coefficients by Louis Cyr and Kennon Francis, 1992) with the following thresholds for interpretation: <0.20 as slight, 0.21–0.40 as fair, 0.41–0.60 as moderate, 0.61–0.80 as substantial, and >0.81 as almost perfect agreement.

For continuous data, we reported median, interquartile range and total range. For dichotomous outcomes, we reported frequencies and percentages. We excluded independent variables with less than 20 observations per value from inferential statistics. We log transformed non-normally distributed continuous variables. We performed a bivariate logistic regression for each eligible independent variable, adjusting for the maximum length of follow-up. We reported our findings using odds ratios (OR), 95% confidence intervals (CI) and p values. Data permitting, we planned a multivariable analysis including adjustment for the study effect. We performed a Fisher's Exact test between statistically significant predictors of outcome to determine the strength of association between these variables. We set the alpha level for statistical significance at 0.05.

## Results

### Individual study and overall estimates

**Figure 1:**
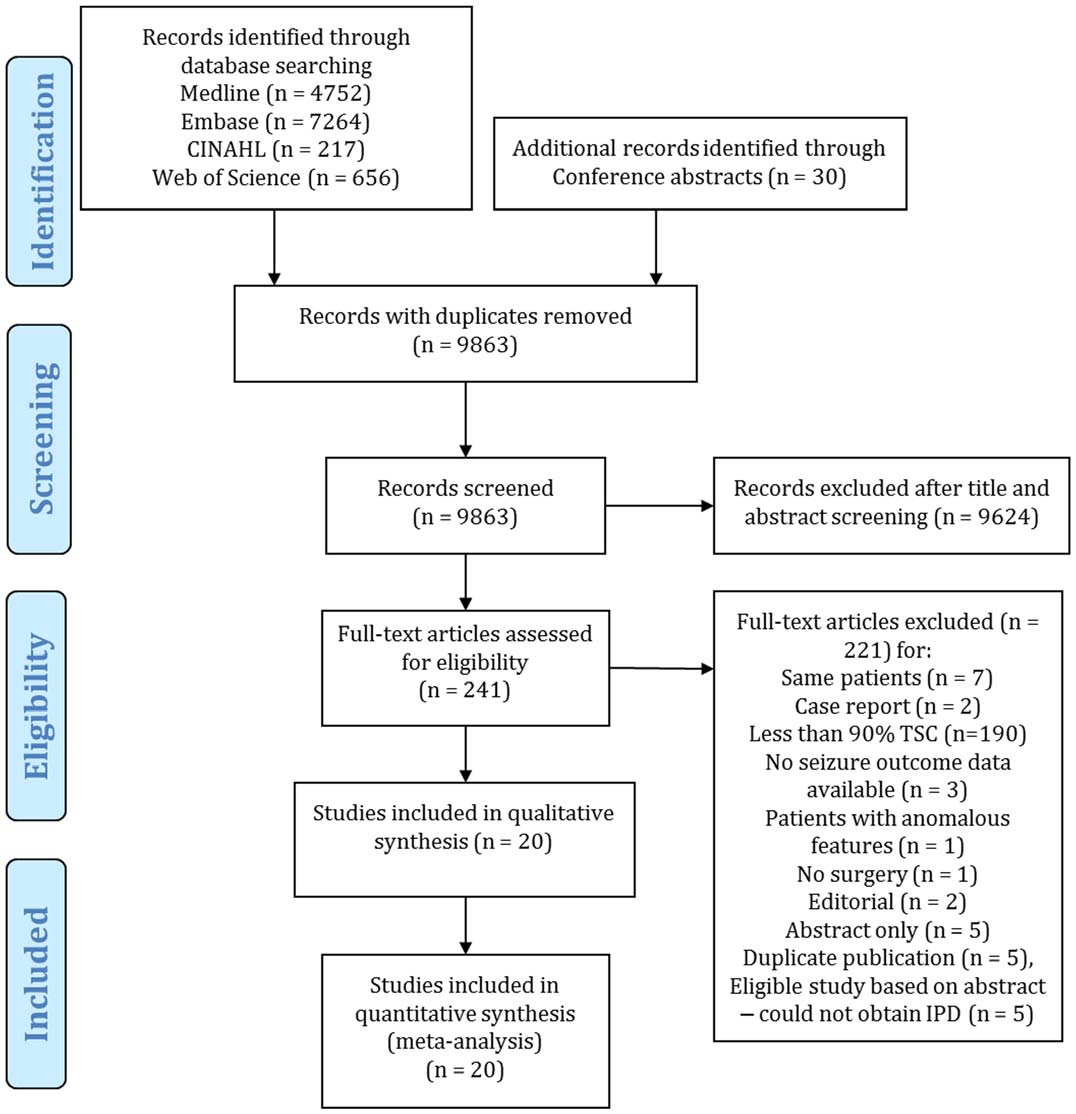
PRISMA 2009 Flow Diagram.

We identified 9863 citations from our electronic database search of Medline, Embase, CINAHL and Web of Science with duplicates removed (Figure 1). We identified 30 additional citations after reviewing conference abstracts. We reviewed 241 articles in full text (unweighted Kappa  = 0.55; 95% CI 0.50–0.60; moderate strength of agreement). We included 20 articles reporting on 185 participants (181 participants had seizure outcome data and were used in the meta-analysis). Appendix S3 presents the excluded articles after full text review (with reasons for exclusion).

**Figure 2:**
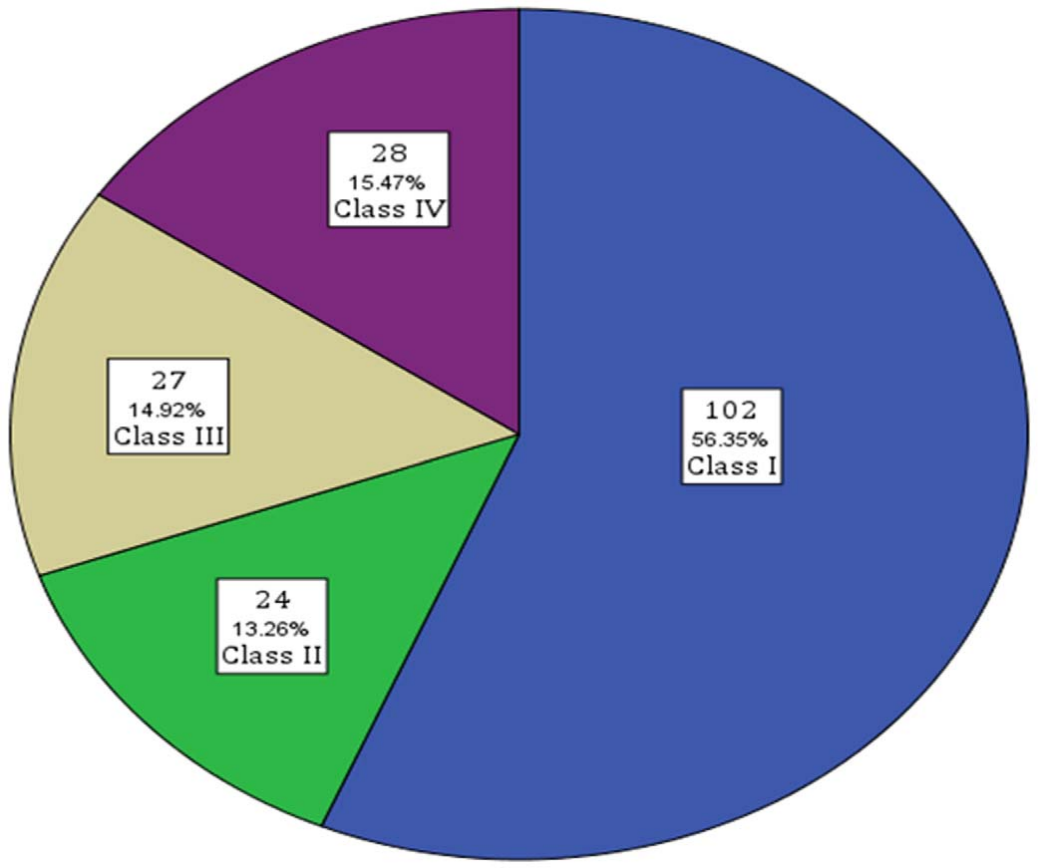
Number and percentage by Engel Classification of participants with TSC undergoing resective epilepsy surgery.

We obtained IPD in 20 of the eligible 25 articles (80%) [11], [12], [16]–[33]. This includes 3 of 8 (38%) articles comprising of 32 participants that we obtained IPD after contacting the corresponding authors. One hundred and twenty-six (70%) of participants achieved a good surgical outcome (i.e. Engel Class I or II) (Figure 2). The median duration of follow-up was 2.3 years (IQR = 1.3–4.3). Table 1 and 2 present the summary descriptive statistics for all independent variables. We excluded size of predominant tuber, invasive interictal and ictal evaluation, PET, SPECT and MEG findings due to the low frequency of observations per value (n<20).

**Table 1:** Frequency table of dichotomous predictors of seizure outcome.

Independent variable	Frequency	Frequency
Gender	52(48.1%) Female	56(51.9%) Male
	**Frequency (Percentage) No**	**Frequency (Percentage) Yes**
Infantile spasms	79(69.9%)	34(30.1%)
Generalized seizures	49(59.8%)	33(40.2%)
Developmental Delay	34(57.6%)	25(42.4%)
EEG/MRI concordance	31(38.8%)	49(61.3%)
	**Frequency (Percentage) Unifocal**	**Frequency (Percentage) Multifocal/Generalized**
Interictal EEG	68(53.5%)	59(46.5%)
Ictal EEG	69(65.1%)	37(34.9%)
PET	-	-
SPECT	5(41.7%)	7(58.3%)
MEG	-	-
Invasive interictal EEG	24(70.6%)	10(29.4%)
Invasive ictal EEG	26(65.0%)	14(35.0%)

**Table 2:** Summary table for continuous predictors of seizure outcome.

Independent variable	N	Median (IQR)	Range
Age at first seizure (months)	126	8.0 (2.0–28.5)	0–216
Preoperative seizure frequency (per day)	30	1.0 (0.0–5.0)	0–35
Age at surgery (years)	174	7.0 (3.0–14.0)	0–46
IQ	28	77.5 (70.25–84.50)	48–119
Size of predominant tuber	-	-	-
Tuber burden	74	5.0 (1.0–15.0)	1–37

### Descriptive information for each study included

Table 3 presents the characteristics of the included studies. Table 4 presents the risk of bias.

**Table 3:** Characteristics of included studies.

First author (year)	No. of patients	Age at surgery range (years)	Study Location	Predictors reported*	Type of surgery performed	Range of follow-up duration (y)	Seizure free
**Aboian (2011)**	6	0.7–13.0	USA	b, d, e, f, h, i, j, k, l	Tuberectomy/lobectomy Multilobar resection	2.0–9.5	3/6(50%)
**Asano (2000)**	7	1.1–9.4	USA	a, d, e, f, i, j, l	*N/A*	0.3–2.3	5/7 (71%)
**Avellino (1997)**	8	3.0–46.0*	USA	a, b, e, f, h, i, k, l	Tuberectomy/lobectomy Multilobar resection	0.7–10.6	4/8 (50%)**
**Baumgartner (1997)**	4	5.0–13.0	USA	a, b, c, d, e, f, h, i, j, k, l, o, p	Tuberectomy/lobectomy Multilobar resection	1.0–11.0	1/4(25%)
**Bebin (1993)**	7	0.8–19.7	USA	d, e, f, l	Tuberectomy/lobectomy	0.8–6.0	5/7(71%)
**Guerreiro (1998)**	11	1.7–54.0	Canada	a, b, d, e, f, g, h, I, j, k, l	Tuberectomy/lobectomy Multilobar resection	0.1–47.0	8/11(67%)**
**Heide (2010)**	6	2.0–29.0	Netherlands	b, c, d, e, f, i, j, k, l	Tuberectomy/lobectomy Multilobar resection	2.8–7.0	4/6(67%)
**Jansen (2006)**	3	6.0–20.0	Netherlands	b, c, d, e, f, g, h, i, l, n, o	*N/A*	2.0–4.0	2/3(67%)
**Jansen (2007)**	6	3.0–36.0	Netherlands	b, c, d, e, f, i, j, k, l, n, o	Tuberectomy/lobectomy Multilobar resection	1.2–6.3	4/6(67%)
**Kagawa (2005)**	17	0.3–12.3	USA	a, b, d, f, i, j, k, l, p	Tuberectomy/lobectomyMultilobar resection Hemispherectomy	0.4–4.8	12/17(71%)
**Kamimura (2006)**	3	2.0–24.0*	Japan	a, c, d, e, f, k	*N/A*	3.0 (mean)	3/3(100%)
**Karenfort (2002)**	8	0.5–34.0	Germany	a, b, f, i, j	Tuberectomy/lobectomyMultilobar resection Hemispherectomy	0.5–4.3	7/8(88%)
**Koh (2000)**	11	0.5–7.5	USA	d, e, f, i, j, m	Multilobar resection	0.5–6.8	8/11(73%)**
**Lachhwani (2005)**	17	0.2–31.0	USA	a, b, f, i, j, k, l	Tuberectomy/lobectomyMultilobar resection	1.0–15.0	12/17(71%)
**Liang (2010)** ***	17	6.0–23.0	China	a, b, d, g, h, l	Tuberectomy/lobectomy	1.0–5.0	13/17(76%)
**Major (2009)**	3	3.0–14.0	USA	a, c, d, e, f, h, i, j, k, l	Tuberectomy/lobectomy	0.8–2.3	2/3(67%)
**Perot (1966)**	7	1.7–26.0	Canada	a, b, c, d, e, f, g, h, i	Tuberectomy/lobectomyMultilobar resection	0.5–16.0	3/7(43%)
**Teutonico (2008)** ***	11	7.1 (median)	Italy/USA	b, i, j	Tuberectomy/lobectomy	0.6–14.0	5/11(45%)
**Weiner (2006)**	25	0.6–16.6	USA	f, o, p	*N/A*	0.5–6.0	23/25(92%)
**Wen (2009)** ***	4	8.0–12.5	China	a, b, d, f	*N/A*	1.8–6.0	3/4 (75%)

Predictors reported* a) Gender; b) Age at seizure onset; c) Preoperative seizure frequency; d) Presence of infantile spasms; e) Presence of generalized seizures; f) Age at surgery; g) Preoperative intelligent Quotient score; h) Presence of moderate/severe developmental delay; i) Focal or generalized/multifocal interictal EEG abnormality; j) Focal or generalized/multifocal ictal EEG abnormality; k) Concordant electroencephalographic and radiological studies; l) Tuber burden; m) Focal or multifocal SPECT abnormality; n) Focal or multifocal MEG abnormality; o) Focal or generalized/multifocal interictal invasive EEG abnormality; p) Focal or generalized/multifocal ictal invasive EEG abnormality.

** Remaining participants were lost to follow-up.

*** Author contacted for IPD.

**Table 4:** Assessment of risk of bias in included studies.

First author (year)	Sample representative?	Prognostic variables well defined?	Confidence in assessment of outcome	Was the follow-up adequate?	Was the treatment standardized?
**Aboian (2011)**	Probably yes	Definitely yes	Probably yes	Definitely yes	Probably yes
**Asano (2000)**	Probably yes	Probably yes	Probably yes	Probably yes	Probably yes
**Avellino (1997)**	Definitely yes	Definitely yes	Probably yes	Probably no	Probably yes
**Baumgartner (1997)**	Definitely yes	Probably yes	Probably yes	Probably no	Probably yes
**Bebin (1993)**	Probably yes	Probably no	Probably yes	Probably yes	Probably yes
**Guerreiro (1998)**	Definitely yes	Probably yes	Probably yes	Probably no	Probably no
**Heide (2010)**	Definitely yes	Probably yes	Probably yes	Definitely yes	Probably yes
**Jansen (2006)**	Definitely yes	Probably yes	Probably yes	Definitely yes	Probably yes
**Jansen (2007)**	Definitely yes	Definitely yes	Probably yes	Definitely yes	Probably no
**Kagawa (2005)**	Definitely yes	Definitely yes	Probably yes	Probably no	Probably yes
**Kamimura (2006)**	Definitely yes	Probably yes	Probably yes	Definitely yes	Probably yes
**Karenfort (2002)**	Definitely yes	Definitely yes	Probably yes	Probably no	Probably yes
**Koh (2000)**	Definitely yes	Probably yes	Probably yes	Definitely not	Probably yes
**Lachhwani (2005)**	Definitely yes	Definitely yes	Probably yes	Probably yes	Probably yes
**Liang (2010)**	Definitely yes	Definitely yes	Probably yes	Definitely yes	Definitely yes
**Major (2009)**	Probably yes	Probably no	Probably yes	Probably yes	Probably yes
**Perot (1966)**	Probably no	Probably no	Probably yes	Probably yes	Probably no
**Teutonico (2008)**	Definitely yes	Definitely yes	Probably yes	Probably yes	Probably yes
**Weiner (2006)**	Definitely yes	Definitely yes	Probably yes	Probably no	Definitely yes
**Wen (2009)**	Definitely yes	Probably yes	Probably yes	Definitely yes	Probably yes

### Statistically significant predictors of outcome

We log transformed age at seizure onset, preoperative seizure frequency and age at first surgery to normalize the distribution. Table 5 presents the OR, 95% confidence interval and p-value for each variable from our logistic regression analyses. Due to the small sample sizes of individual studies (median: 7; range 3–25 patients), and the variable inclusion of predictors across studies, we were unable to conduct a multivariable analysis or adjust for study effects. Statistically significant predictors of good seizure outcomes following resective epilepsy surgery in TSC included absence of generalized seizure semiology (OR = 3.1; 95% CI = 1.2–8.2, p = 0.022), no or mild developmental delay (OR = 7.3; 95% CI = 2.1–24.7, p = 0.001), unifocal ictal scalp EEG abnormality (OR = 3.2; 95% CI = 1.4–7.6, p = 0.008) and EEG/MRI concordance (OR = 4.9; 95% CI = 1.8–13.5, p = 0.002).

**Table 5:** Odds ratios, 95% confidence intervals and p values for preoperative predictors of good seizure outcome adjusted for duration of follow-up.

Independent variable	OR	Lower 95% CI	Higher 95% CI	P value
Gender (Female)	1.092	0.481	2.475	0.834
Log_10_(Age at seizure onset)	1.520	0.772	2.993	0.226
Log_10_(Preoperative seizure frequency)	2.295	0.340	15.512	0.394
Lack of infantile spasms	1.184	0.492	2.849	0.707
Lack of generalized seizures*	3.111	1.175	8.237	0.022
Log_10_ (Age at surgery)	1.211	0.560	2.617	0.626
Preoperative IQ	1.008	0.940	1.081	0.823
No or mild developmental delay*	7.285	2.145	24.739	0.001
No or unifocal interictal scalp EEG abnormality	1.538	0.726	3.257	0.260
Unifocal ictal scalp EEG abnormality*	3.205	1.351	7.576	0.008
Less tuber burden	1.011	0.958	1.068	0.684
EEG/MRI concordance*	4.882	1.763	13.522	0.002

* Statistically significant predictors of postoperative seizure outcome.

### Strength of association amongst statistically significant predictors of outcome

Unifocal ictal EEG abnormality and EEG/MRI concordance had a positive association (Pearson correlation  = 0.37; p = 0.006). Of 43 participants with unifocal ictal EEG abnormality, 32 (74%) had EEG/MRI concordance while from 22 participants with multifocal/generalized EEG abnormality, only 8 (36%) had EEG/MRI concordance. The other variables were not statistically significantly associated with one another.

### Publication bias

Because of the very small number of participants per study, we could not assess between-study heterogeneity or publication bias.

### Assessment of quality of studies

Generally, the studies had low risk of bias with respect to sample representativeness and outcome assessment. There was moderate risk of bias with respect to well defining prognostic variables, adequacy of length of follow-up and standardization of treatment (Table 4).

## Discussion

In our review of 20 studies, 56% of participants with TSC undergoing resective epilepsy surgery achieved Engel Class I outcomes and another 13% Engel Class II outcomes. We identified absence of generalized seizure semiology, no or mild developmental delay, unifocal scalp EEG abnormality and EEG/MRI concordance as predictive factors of good seizure outcome. EEG/MRI concordance may have limited relevance as the tubers are increased. In cases of many (multilobar) lesions, some may coincide with EEG abnormalities without MRI providing useful information. This factor is likely driven by focal EEG abnormalities, rather than MRI.

### Strengths and limitations

Strengths of this review include: 1) We developed our study protocol in advance of conducting the review; 2) We performed a comprehensive search; 3) We did not exclude studies based on language of publication or date of publication; 4) We obtained individual data and performed an IPD meta-analysis; and 5) We adjusted for the length of follow-up therefore eliminating bias that would have resulted if putative predictive variables were associated with length of follow-up.

The review also has limitations: 1) Although an exhaustive search strategy was utilized, it is possible that some studies were not identified due to inappropriate indexing or errors in screening; 2) Non-standardized reporting affects the validity of data abstraction and assessment of risk of bias; 3) There is a lack of recognized criteria for assessment of bias in prognostic cohort studies. This required us to develop and utilize our own instrument which was not validated; 4) Criteria for surgical acceptability as (a worthwhile chance of seizure freedom) may have differed between centers and co-varied with predictive factors; 5) The data did not allow us to perform multivariable regression to ascertain whether variables were independently predictive. Given these limitations, cautious interpretation is required in applying the findings of this study; and 6) Neuropsychological, psychosocial, quality of life and psychiatric outcomes following resective epilepsy surgery which are other patient-important outcome measures were not evaluated.

### Appropriateness of studies assembled for assessing the hypothesis

A requirement for reliable prognostic studies is to choose a cohort of participants that are relatively similar with respect to stage of their disease. In this study, selecting participants who are medically refractory and have undergone resective epilepsy surgery satisfies this criterion. However, this excludes an important group of patients: those with medically refractory epilepsy who are deemed to be poor candidates for resective surgery who either undergo a palliative surgical procedure or do not undergo surgery; some of these participants, although rare, may still result in a good seizure outcome. It is important to note that the participants we identified are likely deemed to have at least a ‘worthwhile’ chance to have a satisfactory seizure outcome after surgery. To the extent that criteria for resectability differ across centers could compromise the generalizability of the results.

### Assessment of confounders

There are two potential sources of confounders:

1. Determination of surgical candidacy and operative plan: The determination of surgical candidacy, technique and risks of surgery is a complex process requiring multidisciplinary collaboration. The groups' recommendation is presented to the child and/or parent who must ultimately be willing to accept the risks of the proposed operation in hopes of attaining a good seizure outcome. The intricacies of management decision, although based on similar principles of epilepsy surgery, are variable at each center, influenced by individual patient values and preferences, and too complex to be accurately captured from retrospective observational studies.
2. Degree of resection of the EZ: an extensive resection of the EZ is associated with better seizure outcomes than a subtotal resection of the EZ. Degree of resection was difficult to ascertain accurately as it was not consistently reported in studies. However, it is reasonable to assume that an extensive resection of the EZ was performed in almost all cases for the following reasons: 1) it is not justifiable to plan a subtotal resection of the EZ if the preoperative goal is attaining seizure freedom; and 2) the vast majority of surgical cases go according to plan. However, we excluded cases that were documented to not have had a complete surgical resection of the hypothesized EZ from this review.

The systematic review by Jansen et al. of 170 participants also reported on IPD obtained from full-text review of the articles. However they did not contact authors when IPD was not available. Although 10 articles overlapped with this review, the participant composition is different as we selectively chose participants within articles to be included in the review. For example, they included participants that have undergone a corpus callosotomy [11] or have a pathological diagnosis other than Tuber [21]. They have also excluded participants from studies for unclear reasons [19]. Similar to this review, they also identified that moderate or severe developmental delay was associated with poor seizure outcomes [4]. The estimated point estimate for no or mild developmental delay predicting a good seizure outcome was lower: OR = 4.1 (Jansen et al.) vs. OR = 7.3 (current study). However in contrast to our review, the authors found no statistically significant difference with respect to generalized seizure semiology and unifocal scalp EEG abnormalities and seizure outcome [4]. Additionally, the authors did not evaluate EEG/MRI concordance as a potential predictor of seizure outcome [4].

## Conclusion

### Consideration of alternative explanations for observed results

The variables we identified and the associated odds ratios, may represent an accurate accounting of the optimal variables for predicting response to surgery. There may, however, be other variables that where either not collected or not well-reported in the studies that may be more powerful variables associated with success. We may also have been misled by the play of chance. Dealing with the former limitation requires each relevant study to collect data on all possible predictors; dealing with both limitations requires a larger sample size.

Given our study's limitations, it may be unwise to use this data in isolation to counsel patients against undertaking epilepsy surgery. However, serious consideration against resective epilepsy surgery should be made in a situation where there are multiple negative predictors of seizure outcome present.

There are two main novel aspects in our study. Firstly, our methodology is the first to employ an IPD meta-analysis approach, which as we discuss is a very robust and rigorous method for conducting meta-analyses. In-and-of-itself, this is very important, especially when considering epilepsy surgery for TSC given that the majority of studies are small with a fair amount of heterogeneity. Secondly, as a result of this scientifically rigorous approach, we have identified novel predictors of seizure outcomes in children with TSC undergoing resective epilepsy surgery. Further, our findings can serve as a platform for hypothesis testing in future multicenter observational studies. The identification of such predictors may inform clinical decision-making and managing patient expectations.

### Guidelines for future research

There are several important challenges in identification of predictors of seizure outcomes in children who undergo resective epilepsy surgery. The two most important are: 1) Rare incidence of disease; and 2) Variability between centers in determining epilepsy surgery candidacy. A large long-term, prospective multicenter observational study is warranted to further evaluate predictors of seizure outcomes. Given the variability in available technology for selecting surgical candidates, experience of the epilepsy team and the epilepsy surgeon, the determination of surgical candidacy and the operative plan must be centrally adjudicated and be statistically adjusted for factors that cannot be controlled across centers. The results of this study and consultation with experts can inform selection of predictors for future studies.

## Supporting Information

Appendix S1 Search strategy for MEDLINE, Embase, CINAHL and Web of Science.
(DOCX)

Appendix S2 Tool to assess risk of bias in prognostic cohort studies.
(DOCX)

Appendix S3 List of excluded articles with reasons.
(DOCX)

Table S1 Participant level data collection.
(DOCX)
