# Supplementary material for: Predictors of Seizure Outcomes in Children with Tuberous Sclerosis Complex and Intractable Epilepsy Undergoing Resective Epilepsy Surgery: An Individual Participant Data Meta-Analysis
Source: PLoS One. 2013 Feb 6;8(2):e53565. doi: 10.1371/journal.pone.0053565 (PMC3566144; doi:10.1371/journal.pone.0053565)
Supplement: Appendix S1 — Search strategy for MEDLINE, Embase, CINAHL and Web of Science. (DOCX) [file pone.0053565.s001.docx]

**Appendix S1**

**Embase** <1980 to 2011 Week 41> (7264 citations)

**Ovid MEDLINE**(R) In-Process & Other Non-Indexed Citations and Ovid MEDLINE(R) <1948 to Present> (4752 citations)

Search performed: October 15, 2011

1. exp Neurosurgical Procedures/

2. surg*.mp.

3. neurosurgery/

4. su.fs.

5. 1 or 3

6. 2 or 4

7. exp Epilepsy/

8. Epilepsy*.mp.

9. 7 or 8

10. tuberous*.mp.

11. 9 and 10

12. child*.mp.

13. infant*.mp.

14. teen*.mp.

15. toddler*.mp.

16. adolescent*.mp.

17. preschool*.mp.

18. 12 or 13 or 14 or 15 or 16 or 17

19. 5 or 6

20. 10 and 19

21. 9 and 18 and 19

22. 20 or 21

23. limit 22 to (article or conference abstract or conference paper or journal or report or case reports or classical article or clinical conference or comparative study or congresses or "corrected and republished article" or duplicate publication or english abstract or evaluation studies or historical article or journal article or multicenter study or retracted publication or twin study)

**CINAHL** (217 citations)

Search performed: October 16, 2011

1. (MM "Neurosurgery+") OR "neurosurgery"

2. (MH "Epilepsy+") OR "epilepsy" OR (MM "Epilepsy, Partial, Complex") OR (MM "Epilepsy, Temporal Lobe") OR (MM "Epilepsy, Partial, Focal") OR (MM "Epilepsy, Partial")

3. s1 and s2

**Web of Science** (656 citations)

Search performed: October 16, 2011

# 3 [656](http://apps.webofknowledge.com.libaccess.lib.mcmaster.ca/summary.do?product=WOS&doc=1&qid=6&SID=1FIb55miJObNkPhFk1J&search_mode=CombineSearches)

#2 AND #1

Databases=SCI-EXPANDED, SSCI, A&HCI, CPCI-S, CPCI-SSH Timespan=All Years

Lemmatization=On

# 2 [77,419](http://apps.webofknowledge.com.libaccess.lib.mcmaster.ca/summary.do?product=WOS&doc=1&qid=3&SID=1FIb55miJObNkPhFk1J&search_mode=GeneralSearch)

Topic=(epilepsy/)

Databases=SCI-EXPANDED, SSCI, A&HCI, CPCI-S, CPCI-SSH Timespan=All Years

Lemmatization=On

# 1 [11,489](http://apps.webofknowledge.com.libaccess.lib.mcmaster.ca/summary.do?product=WOS&doc=1&qid=1&SID=1FIb55miJObNkPhFk1J&search_mode=GeneralSearch)

Topic=(neurosurgery/)

Databases=SCI-EXPANDED, SSCI, A&HCI, CPCI-S, CPCI-SSH Timespan=All Years

Lemmatization=On
