# Supplementary material for: Predictors of Seizure Outcomes in Children with Tuberous Sclerosis Complex and Intractable Epilepsy Undergoing Resective Epilepsy Surgery: An Individual Participant Data Meta-Analysis
Source: PLoS One. 2013 Feb 6;8(2):e53565. doi: 10.1371/journal.pone.0053565 (PMC3566144; doi:10.1371/journal.pone.0053565)
Supplement: Appendix S2 — Tool to assess risk of bias in prognostic cohort studies. (DOCX) [file pone.0053565.s002.docx]

**Appendix S3.**

1. **Was the sample of patients representative?**

Definitely yes Probably yes Probably no Definitely no

(low risk of bias) (high risk of bias)

Examples of low risk of bias:

Definitely yes: Inclusion criteria is well-defined, sample selection is explained and is clear that it is consecutive. Clinical and demographic characteristics fully described and sample is representative.

Probably yes: Inclusion criteria is well-defined, sample selection is partially explained and is unclear that it is consecutive. Clinical and demographic characteristics are partially described and sample is likely representative.

Examples of high risk of bias:

Probably no: Inclusion criteria is poorly defined, sample selection is partially explained and it unclear that it is consecutive. Clinical and demographic characteristics are partially described and sample is likely not representative.

Definitely no: Inclusion criteria is poorly defined, sample selection is not explained and is clearly non-consecutive. Clinical and demographic characteristics are poorly described and sample is not very representative.

**2. Were the prognostic variables well defined and well characterized?**

Definitely yes Probably yes Probably no Definitely no

(low risk of bias) (high risk of bias)

Examples of low risk of bias:

Definitely yes: The prognostic variables are well-defined, including all the details of its measurement, are precisely measured, and available for all patients.

Probably yes: The prognostic variables are well-defined, including some of the details of its measurement, are precisely measured, and available for a high proportion of patients.

Examples of high risk of bias:

Probably no: The prognostic variables are poorly defined, with very few details regarding its measurements, are measured poorly and are available for a low proportion of patients.

Definitely no: The prognostic variables are poorly defined, lack of details regarding its measurements, are measured poorly and are not available for any of the patients.

**3. Can we be confident in the assessment of outcome?**

Definitely yes Probably yes Probably no Definitely no

(low risk of bias) (high risk of bias)

Examples of low risk of bias:

Definitely yes: Outcome is less subjective, fully defined and appropriate. E.g. Parents were asked to keep a seizure diary, follow-up appointments were frequent and the data was collected independently and in duplicate.

Probably yes: Outcome is less subjective, reasonably defined and largely appropriate. E.g. No seizure diary was kept but follow-up appointments were routine and the data was collected by one extractor.

Examples of high risk of bias:

Probably no: Outcome is more subjective, inadequately defined and largely inappropriate. E.g. No seizure diary was kept, long duration for follow-up appointments, and the data was collected by one data extractor.

Definitely no: Outcome is more subjective, poorly defined and inappropriate. E.g. No seizure diary was kept, long duration for follow-up appointments, and the data was collected by one data extractor.

4. **Was the follow-up adequate?**

Definitely yes Probably yes Probably no Definitely no

(low risk of bias) (high risk of bias)

Examples of low risk of bias:

Definitely yes: No missing outcome data; Reasons for missing outcome data unlikely to be related to true outcome; Missing outcome data balanced in numbers across different demographic groups, with similar reasons for missing data across groups.

Probably yes: Rare missing outcome data; Reasons for missing outcome data unlikely to be related to true outcome; Missing outcome data almost balanced in numbers across different demographic groups, with similar reasons for missing data across groups.

Examples of high risk of bias:

Probably no: Occasional missing outcome data; Reason for missing outcome data likely to be related to true outcome, with either imbalance in numbers or reasons for missing data across demographic groups. Definitely no: Common missing outcome data; Reason for missing outcome data likely to be related to true outcome, with clear imbalance in numbers and reasons for missing data across demographic groups.

**5. Can we be confident that the treatment was standardized?**

Definitely yes Probably yes Probably no Definitely no

(low risk of bias) (high risk of bias)

Examples of low risk of bias:

Definitely yes: The treatment is described in detail and was standardized across patients. This means there has been no significant change in the philosophy of care for patients (i.e the same epilepsy surgery team, the same diagnostic tools utilized and the same surgeon).

Probably yes: The treatment is described in some detail and was likely standardized across patients. This means there has been very little change in the philosophy of care for patients (i.e the same epilepsy surgery team, the same diagnostic tools utilized and the same surgeon).

Examples of high risk of bias:

Probably no: The treatment is poorly described and was unlikely to be standardized across patients. There have been some changes in the philosophy of care for patients (i.e. a change in the epilepsy surgery team personnel, change in the diagnostic tools utilized and more than one surgeon).

Definitely no: The treatment is not described and was non-standardized across patients. There have been major changes in the philosophy of care for patients (i.e. a change in the epilepsy surgery team personnel, change in the diagnostic tools utilized and more than one surgeon).
