# Supplementary material for: Predictors of Seizure Outcomes in Children with Tuberous Sclerosis Complex and Intractable Epilepsy Undergoing Resective Epilepsy Surgery: An Individual Participant Data Meta-Analysis
Source: PLoS One. 2013 Feb 6;8(2):e53565. doi: 10.1371/journal.pone.0053565 (PMC3566144; doi:10.1371/journal.pone.0053565)
Supplement: Appendix S3 — List of excluded articles with reasons. (DOCX) [file pone.0053565.s003.docx]

**Appendix S4.**

Case reports (2)

1. Tuberous sclerosis and a not so benign astrocytoma*.* United States, 2002, p 316.

2. Bye AM, Matheson JM, Tobias VH, Mackenzie RA: Selective epilepsy surgery in tuberous sclerosis*.* AUSTRALIA, Department of Paediatric Neurology, Prince of Wales Children's Hospital, Randwick, New South Wales, Australia, 1989, pp 243-245.

Patient overlap with another article (7)

1. Aboian MS, Wong-Kisiel LC, Rank M, Wetjen N, Wirrell EC, Witte RJ: Can subtraction ictal SPECT Co-registered to MRI identify epileptogenic foci in children with tuberous sclerosis?, John Wiley and Sons Inc, 2010, p S127.

2. Carlson C, Teutonico F, Elliott RE, Moshel YA, LaJoie J, Miles D, Devinsky O, Weiner HL: Bilateral invasive electroencephalography in patients with tuberous sclerosis complex: A path to surgery? Clinical article*.* United States, American Association of Neurological Surgeons (1224 West Main Street Suite 450, Charlottesville VA 22903, United States), 2011, pp 421-430.

3. Liang S, Zhao M, Yi L, Li A, Sun Y: Epilepsy surgery in tuberous sclerosis complex, John Libbey Eurotext, 2009, p 166.

4. Moshel YA, Elliott R, Teutonico F, Sellin J, Carlson C, Devinsky O, Weiner HL: Do tubers contain function? Resection of epileptogenic foci in perirolandic cortex in children with tuberous sclerosis complex*.* United States, Blackwell Publishing Inc. (350 Main Street, Malden MA 02148, United States), 2010, pp 1242-1251.

5. Oliveira RSD, Volpon MS, Terra VC, Sakamoto AC, Machado HR: Rolandic cortex epilepsy surgery in children: A single 1-center experience with 48 consecutive cases, Lippincott Williams and Wilkins, 2010, p 554.

6. Roth J, Olasunkanmi A, MacAllister WS, Weil E, Uy CC, Devinsky O, Weiner HL: Quality of life following epilepsy surgery for children with tuberous sclerosis complex*.* United States, Academic Press Inc. (6277 Sea Harbor Drive, Orlando FL 32887-4900, United States), 2011, pp 561-565.

7. Teutonico F, Carlson C, Devinsky O, Lajoie J, Miles D, Weiner H: Epilepsy surgery for children with tuberous sclerosis complex and multifocal EEG findings, John Libbey Eurotext, 2009, p 167.

Less than 90% TSC participants (190)

1. Adelson PD, Black Mc LP, Madsen JR, Kramer U, Rockoff MA, Riviello JJ, et al. Use of subdural grids and strip electrodes to identify a seizure focus in children. Switzerland1995. p. 174-80.

2. Adler J, Erba G, Winston KR, Welch K, Lombroso CT. RESULTS OF SURGERY FOR EXTRATEMPORAL PARTIAL EPILEPSY THAT BEGAN IN CHILDHOOD. Archives of Neurology. 1991;48(2):133-40.

3. Ahnlide JA, Rosen I, Linden-Mickelsson TP, Kallen K. Does SISCOM contribute to favorable seizure outcome after epilepsy surgery? United States2007. p. 579-88.

4. Akos SC, Rothner AD, Kotagal P, Erenberg G, Dinner DS, Wyllie E. Symptomatic or cryptogenic partial epilepsy of childhood onset: Fourteen-year follow-up. United States2001. p. 264-9.

5. Asano E, Juhasz C, Shah A, Muzik O, Chugani DC, Shah J, et al. Origin and propagation of epileptic spasms delineated on electrocorticography. United States2005. p. 1086-97.

6. Asano E, Juhasz C, Shah A, Sood S, Chugani HT. Role of subdural electrocorticography in prediction of long-term seizure outcome in epilepsy surgery. Blackwell Publishing Inc; 2009. p. 164-5.

7. Aykut-Bingol C, Bronen RA, Kim JH, Spencer DD, Spencer SS. Surgical outcome in occipital lobe epilepsy: Implications for pathophysiology. United States1998. p. 60-9.

8. Balestri M, Mai R, Castana L, Didato G, Rossi M, Lo RG, et al. Clinical features of a surgical infantile onset focal epileptic population. 2008(Web Page):303-4.

9. Bass N, Wyllie E, Comair Y, Kotagal P, Ruggieri P, Holthausen H. Supplementary sensorimotor area seizures in children and adolescents. United States1995. p. 537-44.

10. Battaglia D, Chieffo D, Lettori D, Perrino F, Di RC, Guzzetta F. Cognitive assessment in epilepsy surgery of children. Germany2006. p. 744-59.

11. Bauman JA, Feoli E, Romanelli P, Doyle WK, Devinsky O, Weiner HL. Multistage epilepsy surgery: Safety, efficacy, and utility of a novel approach in pediatric extratemporal epilepsy. United States2005. p. 318-32.

12. Beardsworth ED, Zaidel DW. Memory for faces in epileptic children before and after brain surgery. Netherlands1994. p. 589-96.

13. Beckung E, Uvebrant P, Hedstrom A, Rydenhag B. The effects of epilepsy surgery on the sensorimotor function of children. United Kingdom1994. p. 893-901.

14. Behdad A, Limbrick DD, Jr., Bertrand ME, Smyth MD. Epilepsy surgery in children with seizures arising from the rolandic cortex. United States: Department of Pathology, Cedars-Sinai Medical Center, Los Angeles, California 63110, USA; 2009. p. 1450-61.

15. Behrens E, Schramm J, Zentner J, Konig R. Surgical and neurological complications in a series of 708 epilepsy surgery procedures. United States1997. p. 1-10.

16. Benifla M, Otsubo H, Ochi A, Weiss SK, Donner EJ, Shroff M, et al. Temporal lobe surgery for intractable epilepsy in children: An analysis of outcomes in 126 children. United States2006. p. 1203-13.

17. Benifla M, Rutka JT, Otsubo H, Lamberti-Pasculli M, Elliott I, Sell E, et al. Long-term seizure and social outcomes following temporal lobe surgery for intractable epilepsy during childhood. Netherlands: Elsevier (P.O. Box 211, Amsterdam 1000 AE, Netherlands); 2008. p. 133-8.

18. Bidzinski J, Bacia T, Ruzikowski E. The results of the surgical treatment of occipital lobe epilepsy. AUSTRIA: Department of Neurosurgery, Medical Academy, Warsaw, Poland; 1992. p. 128-30.

19. Bittar RG, Rosenfeld JV, Klug GL, Hopkins IJ, Simon HA. Resective surgery in infants and young children with intractable epilepsy. United Kingdom2002. p. 142-6.

20. Bizzi JWJ, Bruce DA, North R, Elterman R, Linder S, Porter-Levy S, et al. Surgical treatment of focal epilepsy in children: Results in 37 patients. Switzerland1997. p. 83-92.

21. Blackburn LB, Lee GP, Westerveld M, Hempel A, Park YD, Loring DW. The Verbal IQ/Performance IQ discrepancy as a sign of seizure focus laterality in pediatric patients with epilepsy. United States2007. p. 84-8.

22. Bleasel A, Kotagal P, Kankirawatana P, Rybicki L. Lateralizing value and semiology of ictal limb posturing and version in temporal lobe and extratemporal epilepsy. UNITED STATES: Section of Epilepsy & Sleep Disorders, Cleveland Clinic Foundation, OH 44195, USA; 1997. p. 168-74.

23. Blum DE, Ehsan T, Dungan D, Karis JP, Fisher RS. Bilateral temporal hypometabolism in epilepsy. United States1998. p. 651-9.

24. Blume WT, Girvin JP, McLachlan RS, Gilmore BE. Effective temporal lobectomy in childhood without invasive EEG. UNITED STATES: University Hospital, University of Western Ontario, London, Canada; 1997. p. 164-7.

25. Blume WT, Kaibara M. Localization of epileptic foci in children. CANADA: Epilepsy Unit, University Hospital, University of Western Ontario, London, Canada; 1991. p. 570-2.

26. Bocti C, Robitaille Y, Diadori P, Lortie A, Mercier C, Bouthillier A, et al. The pathological basis of temporal lobe epilepsy in childhood. United States2003. p. 191-5.

27. Boshuisen K, Braams O, Jennekens-Schinkel A, Braun KP, Jansen FE, van RP, et al. Medication Policy After Epilepsy Surgery. United States: Elsevier Inc. (360 Park Avenue South, New York NY 10010, United States); 2009. p. 332-8.

28. Boshuisen K, Uiterwaal CSPM, Van NO, Braun KPJ. The TimeToStop study I. Antiepileptic drug withdrawal policies after childhood epilepsy surgery in Europe. Blackwell Publishing Inc; 2010. p. 42-3.

29. Bourgeois M, Di RF, Roujeau T, Boddaert N, Lelouch-Tubiana A, Varlet P, et al. Epilepsy and focal lesions in children. Surgical management. France2008. p. 362-5.

30. Bourgeois M, Sainte-Rose C, Lellouch-Tubiana A, Malucci C, Brunelle F, Maixner W, et al. Surgery of epilepsy associated with focal lesions in childhood. United States1999. p. 833-42.

31. Bye AME, Foo S. Complex partial seizures in young children. United States1994. p. 482-8.

32. C:son Silander H, Blom S, Malmgren K, Rosen I, Uvebrant P. Surgical treatment for epilepsy: a retrospective Swedish multicenter study. DENMARK: Department of Neurosurgery, Uppsala University Hospital, Sweden; 1997. p. 321-30.

33. Cabourne E, Morrall M, Gayatri N. Audit of the leeds regional paediatric epilepsy surgery programme: Analysis of investigations and outcomes. Blackwell Publishing Ltd; 2009. p. 26-7.

34. Caplan R, Guthrie D, Komo S, Donald SW, Sigmann M. Infantile spasms: The development of nonverbal communication after epilepsy surgery. Switzerland1999. p. 165-73.

35. Caplan R, Guthrie D, Komo S, Shields WD, Sigman M. Infantile spasms: Facial expression of affect before and after epilepsy surgery. United States1999. p. 116-32.

36. Carson BSS. Indications and outcomes for lobectomy, corpus callosotomy, and hemispherectomy in pediatric neurosurgical patients. United States: Johns Hopkins Medical Institutions, Baltimore, Maryland, USA; 2000. p. 385-99.

37. Cascino GD, Jack CRJ, Parisi JE, Marsh WR, Kelly PJ, Sharbrough FW, et al. MRI in the presurgical evaluation of patients with frontal lobe epilepsy and children with temporal lobe epilepsy: pathologic correlation and prognostic importance. NETHERLANDS: Epilepsy Service, Mayo Clinic, Rochester, MN; 1992. p. 51-9.

38. Cascino GD, Trenerry MR. Significance of postoperative electroencephalograms in patients with extratemporal lesional epilepsy. United States1994. p. 2-6.

39. Chandler CL, Polkey CE. Epilepsy surgery in children: 17 years experience. United Kingdom1997. p. 459-60.

40. Chandra S, Dagar A, Chaudhary K, Chauhan A, Bal C, Garg A, et al. To assess the children operated for drug resistant epilepsy from a large tertiary care center in India. Blackwell Publishing Inc; 2011. p. 188.

41. Chugani HT, Juhasz C, Chugani DC, Lawrenson L, Muzik O, Chakraborty PK, et al. Increased striatal serotonin synthesis following cortical resection in children with intractable epilepsy. Netherlands2008. p. 124-30.

42. Chugani HT, Kumar A, Kupsky W, Asano E, Sood S. Clinical and histopathological correlates of 11C-alpha-methyl-L-tryptophan (AMT) PET abnormalities in children with intractable epilepsy. Blackwell Publishing Inc; 2009. p. 435-6.

43. Chugani HT, Shewmon DA, Shields WD, Sankar R, Comair Y, Vinters HV, et al. Surgery for intractable infantile spasms: Neuroimaging perspectives. United States1993. p. 764-71.

44. Ciganek L, Benko J. Experiences with surgical therapy of epilepsy in children. Germany1970. p. 196-200.

45. Clusmann H, Kral T, Gleissner U, Sassen R, Urbach H, Blumcke I, et al. Analysis of Different Types of Resection for Pediatric Patients with Temporal Lobe Epilepsy. United States2004. p. 847-60.

46. Cossu M, Lo RG, Francione S, Mai R, Nobili L, Sartori I, et al. Epilepsy surgery in children: Results and predictors of outcome on seizures. United States2008. p. 65-72.

47. Daniel RT, Meagher-Villemure K, Farmer JP, Andermann F, Villemure JG. Posterior quadrantic epilepsy surgery: Technical variants, surgical anatomy, and case series. United States2007. p. 1429-37.

48. Danielsson S, Rydenhag B, Uvebrant P, Nordborg C, Olsson I. Temporal lobe resections in children with epilepsy: Neuropsychiatric status in relation to neuropathology and seizure outcome. United States2002. p. 76-81.

49. Duchowny M, Jayakar P, Resnick T, Harvey AS, Alvarez L, Dean P, et al. Epilepsy surgery in the first three years of life. United States1998. p. 737-43.

50. Duchowny M, Jayakar P, Resnick T, Levin B, Alvarez L. Posterior temporal epilepsy: Electroclinical features. United States1994. p. 427-31.

51. Duchowny M, Levin B, Jayakar P, Resnick T, Alvarez L, Morrison G, et al. TEMPORAL LOBECTOMY IN EARLY-CHILDHOOD. Epilepsia. 1992;33(2):298-303.

52. Duncan JD, David MS, Bandy DJ, Manwaring K, Kaplan AM, Reiman EM, et al. Use of positron emission tomography for presurgical localization of eloquent brain areas in children with seizures. Switzerland1997. p. 144-56.

53. Dunkley C, Kung J, Scott RC, Nicolaides P, Neville B, Aylett SE, et al. Epilepsy surgery in children under 3 years. Netherlands: Elsevier (P.O. Box 211, Amsterdam 1000 AE, Netherlands); 2011. p. 96-106.

54. Elliott IM, Lach L, Kadis DS, Smith ML. Psychosocial outcomes in children two years after epilepsy surgery: Has anything changed? United States2008. p. 634-41.

55. Ferrier CH, Engelsman J, Alarcon G, Binnie CD, Polkey CE. Prognostic factors in presurgical assessment of frontal lobe epilepsy. ENGLAND: Institute of Epileptology, King's College Hospital, London, UK; 1999. p. 350-6.

56. Fish DR, Smith SJ, Quesney LF, Andermann F, Rasmussen T. Surgical treatment of children with medically intractable frontal or temporal lobe epilepsy: Results and highlights of 40 years' experience. United States1993. p. 244-7.

57. Flanagan D, Valentin A, Garcia SJ, Alarcon G, Boyd SG. Single-pulse electrical stimulation helps to identify epileptogenic cortex in children. United States: Blackwell Publishing Inc. (350 Main Street, Malden MA 02148, United States); 2009. p. 1793-803.

58. Fohlen M, Jalin C, Bulteau C, Delalande O. Surgical treatment of epilepsy in children younger than 3 years of age. France2002. p. 87s-9s.

59. Frater JL, Prayson RA, Morris IH, Bingaman WE. Surgical pathologic findings of extratemporal-based intractable epilepsy: A study of 133 consecutive resections. United States2000. p. 545-9.

60. Garcia-Flores E. Surgical treatment of complex partial seizures: 20 Years' experience. Switzerland1994. p. 216-21.

61. Gashlan M, Loy-English I, Ventureyra ECG, Keene D. Predictors of seizure outcome following cortical resection in pediatric and adolescent patients with medically refractory epilepsy. Germany1999. p. 45-51.

62. Gelinas JN, Battison AW, Smith S, Connolly MB, Steinbok P. Electrocorticography and seizure outcomes in children with lesional epilepsy. Germany: Springer Verlag (Tiergartenstrasse 17, Heidelberg D-69121, Germany); 2011. p. 381-90.

63. Gilliam F, Wyllie E, Kashden J, Faught E, Kotagal P, Bebin M, et al. Epilepsy surgery outcome: comprehensive assessment in children. UNITED STATES: Department of Neurology, University of Alabama at Birmingham, 35294, USA; 1997. p. 1368-74.

64. Gleissner U, Clusmann H, Sassen R, Elger CE, Helmstaedter C. Postsurgical outcome in pediatric patients with epilepsy: A comparison of patients with intellectual disabilities, subaverage intelligence, and average-range intelligence. United States2006. p. 406-14.

65. Gleissner U, Kuczaty S, Clusmann H, Elger CE, Helmstaedter C. Neuropsychological results in pediatric patients with epilepsy surgery in the parietal cortex. United States2008. p. 700-4.

66. Gleissner U, Sassen R, Schramm J, Elger CE, Helmstaedter C. Greater functional recovery after temporal lobe epilepsy surgery in children. United Kingdom2005. p. 2822-9.

67. Goldring S. A method for surgical management of focal epilepsy, especially as it relates to children. UNITED STATES1978. p. 344-56.

68. Goldring S. Pediatric epilepsy surgery. United States1987. p. S82-102.

69. Goldring S, Gregorie EM. Surgical management of epilepsy using epidural recordings to localize the seizure focus. Review of 100 cases. United States1984. p. 457-66.

70. Goldstein R, Harvey AS, Duchowny M, Jayakar P, Altman N, Resnick T, et al. Preoperative clinical, EEG, and imaging findings do not predict seizure outcome following temporal lobectomy in childhood. UNITED STATES: Comprehensive Epilepsy Center, University of Miami School of Medicine, FL, USA; 1996. p. 445-50.

71. Grppel G, Dressler A, Freilinger M, Pahs G, Mayer H, Porsche B, et al. Surgery during early life in children with intractable epilepsy - A prospective evaluation. Blackwell Publishing Inc; 2009. p. 39.

72. Guldvog B, Loyning Y, Hauglie-Hanssen E, Flood S, Bjornaes H. Surgical treatment for partial epilepsy among Norwegian children and adolescents. UNITED STATES: Foundation for Health Services Research, Nordbyhagen, Norway; 1994. p. 554-65.

73. Harbord MG, Manson JI. Temporal lobe epilepsy in childhood: reappraisal of etiology and outcome. United States1987. p. 263-8.

74. Hartley LM, Gordon I, Harkness W, Harding B, Neville BGR, Cross JH. Correlation of SPECT with pathology and seizure outcome in children undergoing epilepsy surgery. United Kingdom2002. p. 676-80.

75. Hemb M, Velasco TR, Parnes MS, Wu JY, Lerner JT, Matsumoto JH, et al. Improved outcomes in pediatric epilepsy surgery: The UCLA experience, 1986-2008. United States: Lippincott Williams and Wilkins (530 Walnut Street, Philadelphia PA 19106-3621, United States); 2010. p. 1768-75.

76. Hirfanoglu T, Serdaroglu A, Cansu A, Bilir E. Outcome of prolonged video-EEG monitoring in children with epilepsy. Turkey: Turkiye Klinikleri Journal of Medical Sciences (Talapapa Bulvary no. 102, Hamammonu 1 06230, Turkey); 2010. p. 1566-74.

77. Hirsch LJ, Spencer SS, Spencer DD, Williamson PD, Mattson RH. Temporal lobectomy in patients with bitemporal epilepsy defined by depth electroencephalography. UNITED STATES: Department of Neurology, Yale University School of Medicine, New Haven, CT; 1991. p. 347-56.

78. Hnojcikova M, Nickels KC, Wetjen NM, Buchhalter JR, Raffel C, Wirrell EC. EEG and neuroimaging studies in young children having epilepsy surgery. United States: Masaryk University, Brno, Czech Republic; 2010. p. 335-40.

79. Hoppe C, Poepel A, Sassen R, Elger CE. Discontinuation of anticonvulsant medication after epilepsy surgery in children. United States2006. p. 580-3.

80. Hoshida T, Chitoku S, Goda K, Honda M, Sakaki T. Surgery for intractable epilepsy in children: Pre-and perioperative evaluations with special reference to dipole tracing method estimated from interictal spike. Japan2001. p. 135-44.

81. Huang C, Marsh ED, Litt B, Storm PB, Porter BE. Incomplete resection of the seizure onset zone is compatible with post-operative seizure freedom. John Wiley and Sons Inc; 2010. p. S125.

82. Huber Z. Surgical treatment of clinical syndromes of intractable epilepsy in developmental age. Poland2000. p. 225-32.

83. Hum KM, Elliott IM, Smith ML. Child and adolescent perceptions of social function two years after epilepsy surgery. Blackwell Publishing Inc; 2009. p. 284-5.

84. Hum KM, Smith ML, Lach L, Elliott IM. Self-perceptions of social function 2 years after pediatric epilepsy surgery. United States: Academic Press Inc. (6277 Sea Harbor Drive, Orlando FL 32887-4900, United States); 2010. p. 354-9.

85. Hwang PA, Otsubo H, Koo BK, Gilday DL, Chuang SH, Jay V, et al. Infantile spasms: cerebral blood flow abnormalities correlate with EEG, neuroimaging, and pathologic findings. UNITED STATES: Department of Pediatrics, Hospital for Sick Children, Toronto, Ontario, Canada; 1996. p. 220-5.

86. Iida K, Otsubo H, Matsumoto Y, Ochi A, Oishi M, Holowka S, et al. Characterizing magnetic spike sources by using magnetoencephalography- guided neuronavigation in epilepsy surgery in pediatric patients. United States2005. p. 187-96.

87. Jacobs J, Kerber K, LeVan P, Schulze-Bonhage A, Korinthenberg R. High frequency oscillations as novel markers of epileptogenicity in children with intractable epilepsy. Springer Verlag; 2011. p. 266.

88. Jayalakshmi S, Panigrahi M, Kulkarni D, Uppin M, Somayajula S, Challa S. Outcome of epilepsy surgery in children after evaluation with non-invasive protocol. India: Medknow Publications and Media Pvt. Ltd (A-109, Kanara Business Centre, off Link Road, Ghatkopar (E), Mumbai 400 075, India); 2011. p. 30-6.

89. Juhasz C, Chugani DC, Muzik O, Shah A, Asano E, Mangner TJ, et al. Alpha-methyl-L-tryptophan PET detects epileptogenic cortex in children with intractable epilepsy. United States2003. p. 960-8.

90. Juhasz C, Chugani DC, Muzik O, Shah A, Shah J, Watson C, et al. Relationship of flumazenil and glucose PET abnormalities to neocortical epilepsy surgery outcome. United States2001. p. 1650-8.

91. Kameyama S, Morota N, Fukuda M, Oishi M, Masuda H. Tailored cortical resection for neocortical epilepsy: Preoperative evaluation, indication and outcomes. Japan2002. p. 584-91.

92. Kaminska A, Chiron C, Ville D, Dellatolas G, Hollo A, Cieuta C, et al. Ictal SPECT in children with epilepsy: comparison with intracranial EEG and relation to postsurgical outcome. England: Department of Neuropediatrics, Saint Vincent de Paul Hospital, Paris, France. anna.kaminska@svp.ap-hop-paris.fr; 2003. p. 248-60.

93. Kan P, Orman C, Kestle JRW. Outcomes after surgery for focal epilepsy in children. Germany2008. p. 587-91.

94. Keene DL, Higgins MJ, Ventureyra ECG. Outcome and life prospects after surgical management of medically intractable epilepsy in patients under 18 years of age. Germany1997. p. 530-5.

95. Keene DL, Loy-English I, Ventureyra ECG. Long-term socioeconomic outcome following surgical intervention in the treatment of refractory epilepsy in childhood and adolescence. Germany1998. p. 362-5.

96. Keene DL, Loy-English I, Ventureyra ECG. Patient satisfaction with surgical treatment of refractory epilepsy done in childhood and early adolescence. Germany1998. p. 30-2.

97. Kim H, Lee C, Knowlton R, Rozzell C, Blount JP. Safety and utility of supplemental depth electrodes for localizing the ictal onset zone in pediatric neocortical epilepsy: Clinical article. United States: American Association of Neurological Surgeons (1224 West Main Street Suite 450, Charlottesville VA 22903, United States); 2011. p. 49-56.

98. Kim JT, Bai SJ, Choi KO, Lee YJ, Park HJ, Kim DS, et al. Comparison of various imaging modalities in localization of epileptogenic lesion using epilepsy surgery outcome in pediatric patients. United Kingdom: W.B. Saunders Ltd (32 Jamestown Road, London NW1 7BY, United Kingdom); 2009. p. 504-10.

99. Kim SK, Wang KC, Hwang YS, Ki JK, Jong HC, Kim IO, et al. Epilepsy surgery in children: Outcomes and complications. United States2008. p. 277-83.

100. Kim SK, Wang KC, Hwang YS, Kim KJ, Kim IO, Lee DS, et al. Pediatric intractable epilepsy: The role of presurgical evaluation and seizure outcome. Germany2000. p. 278-86.

101. Korkman M, Granstrom ML, Kantola-Sorsa E, Gaily E, Paetau R, Liukkonen E, et al. Two-year follow-up of intelligence after pediatric epilepsy surgery. United States2005. p. 173-8.

102. Kral T, Kuczaty S, Blumcke I, Urbach H, Clusmann H, Wiestler OD, et al. Postsurgical outcome of children and adolescents with medically refractory frontal lobe epilepsies. Germany2001. p. 595-601.

103. Kuehn SM, Keene DL, Richards PMP, Ventureyra ECG. Are there changes in intelligence and memory functioning following surgery for the treatment of refractory epilepsy in childhood? Germany2002. p. 306-10.

104. Kurian M, Spinelli L, Delavelle J, Willi JP, Velazquez M, Chaves V, et al. Multimodality imaging for focus localization in pediatric pharmacoresistant epilepsy. France2007. p. 20-31.

105. Lach LM, Elliott I, Giecko T, Olds J, Snyder T, McCleary L, et al. Patient-reported outcome of pediatric epilepsy surgery: social inclusion or exclusion as young adults? United States: School of Social Work, McGill University, Montreal, Quebec, Canada; 2010. p. 2089-97.

106. Larkins MV, Hahn JF. Epilepsy surgery in children and young adults. The Cleveland Clinic experience. UNITED STATES1989. p. S266-8.

107. Larysz D, Larysz P, Mandera M. Evaluation of quality of life and clinical status of children operated on for intractable epilepsy. Germany2007. p. 91-7.

108. Laws ERJ, Bertram EH. Epilepsy surgery in children and adolescents. United States: Department of Neurosurgery, University of Virginia Health Sciences Center, Charlottesville, Virginia 22908, USA; 1996. p. e1.

109. Lawson JA, Cook MJ, Vogrin S, Litewka L, Strong D, Bleasel AF, et al. Clinical, EEG, and quantitative MRI differences in pediatric frontal and temporal lobe epilepsy. United States: Sydney Children's Hospital, Department of Paediatric Neurology, University of New South Wales, Randwick, Australia; 2002. p. 723-9.

110. Lee GP, Park YD, Westerveld M, Hempel A, Blackburn LB, Loring DW. Wada memory performance predicts seizure outcome after epilepsy surgery in children. United States2003. p. 936-43.

111. Lee J, Kim JY, Kim HS, Nam SH, Lee M, Chang MS, et al. Cognitive outcome of temporal lobe resection in childhood with temporal lobe epilepsy. Blackwell Publishing Inc; 2011. p. 211.

112. Lee JJ, Kang WJ, Lee DS, Lee JS, Hwang H, Kim KJ, et al. Diagnostic performance of 18F-FDG PET and ictal 99mTc-HMPAO SPET in pediatric temporal lobe epilepsy: quantitative analysis by statistical parametric mapping, statistical probabilistic anatomical map, and subtraction ictal SPET. United Kingdom2005. p. 213-20.

113. Lehtinen H, Gaily E, Korkman M. Neuropsychological outcome in preadolescent children after epilepsy surgery in the temporal lobe. John Libbey Eurotext; 2009. p. 169.

114. Leiphart JW, Peacock WJ, Mathern GW. Lobar and multilobar resections for medically intractable pediatric epilepsy. Switzerland2001. p. 311-8.

115. Lendt M, Gleissner U, Helmstaedter C, Sassen R, Clusmann H, Elger CE. Neuropsychological outcome in children after frontal lobe epilepsy surgery. United States2002. p. 51-9.

116. Lendt M, Helmstaedter C, Elger CE. Pre- and postoperative neuropsychological profiles in children and adolescents with temporal lobe epilepsy. United States1999. p. 1543-50.

117. Lendt M, Helmstaedter C, Kuczaty S, Schramm J, Elger CE. Behavioural disorders in children with epilepsy: Early improvement after surgery. United Kingdom2000. p. 739-44.

118. Li YL, Luan GM, Zhou J, Bao M. Pediatric intractable epilepsy surgery. China2008. p. 210-2.

119. Liava A, Francione S, Tassi L, Lo RG, Cossu M, Mai R, et al. Individually tailored epilepsy surgery in strictly extratemporal localization: Outcome predictors in a population of 37 cases with epilepsy onset under 12 years. Blackwell Publishing Inc; 2011. p. 193.

120. Liu S, An N, Yang H, Yang M, Hou Z, Liu L, et al. Pediatric intractable epilepsy syndromes: Reason for early surgical intervention. Netherlands2007. p. 69-78.

121. Loddenkemper T, Holland KD, Stanford LD, Kotagal P, Bingaman W, Wyllie E. Developmental outcome after epilepsy surgery in infancy. United States2007. p. 930-5.

122. Lou SM, Elliott I, Lach L. Memory outcome after pediatric epilepsy surgery: Objective and subjective perspectives. United Kingdom2006. p. 151-64.

123. Lynch BJ, O'Tuama LA, Treves ST, Mikati M, Holmes GL. Correlation of 99m Tc-HMPAO SPECT with EEG monitoring: Prognostic value for outcome of epilepsy surgery in children. Netherlands1995. p. 409-17.

124. Maehara T, Shimizu H, Oda M, Arai N. Surgical treatment of children with medically intractable epilepsy--outcome of various surgical procedures. JAPAN: Department of Neurosurgery, Tokyo Metropolitan Neurological Hospital; 1996. p. 305-9.

125. Malmgren K, Olsson I, Engman E, Flink R, Rydenhag B. Seizure outcome after resective epilepsy surgery in patients with low IQ. United Kingdom2008. p. 535-42.

126. Malmgren K, Olsson I, Flink R, Rydenhag B. Prospective longitudinal 5- and 10-year seizure outcome of resective epilepsy surgery in Sweden. Blackwell Publishing Inc; 2009. p. 453.

127. Mathern GW, Giza CC, Yudovin S, Vinters HV, Peacock WJ, Shewmon DA, et al. Postoperative seizure control and antiepileptic drug use in pediatric epilepsy surgery patients: The UCLA experience, 1986-1997. United States1999. p. 1740-9.

128. Melikyan A, Arkhipova N, Kazaryan A, Golovteev A, Pronin I, Grinenko O, et al. Pediatric epilepsy surgery in Russia: A 5-year experience from a single institution. Blackwell Publishing Inc; 2011. p. 182.

129. Minassian BA, Otsubo H, Weiss S, Elliott I, Rutka JT, Snead IO. Magnetoencephalographic localization in pediatric epilepsy surgery: Comparison with invasive intracranial electroencephalography. United States1999. p. 627-33.

130. Minotti L, Kahane P. Surgery of epilepsy in children. France2006. p. 325-32.

131. Miserocchi A, Fuschillo D, Schiariti M, Gozzo F, Castana L, Cardinale F, et al. Results of surgery for temporal lobe epilepsy in children. Blackwell Publishing Inc; 2011. p. 183.

132. Mittal S, Montes JL, Farmer JP, Rosenblatt B, Dubeau F, Andermann F, et al. Long-term outcome after surgical treatment of temporal lobe epilepsy in children. United States2005. p. 401-12.

133. Mohamed A, Freeman JL, Bailey CA, Maixner WJ, Harvey AS. Epilepsy surgery in children with tuberous sclerosis, epileptic spasms and multiple seizure foci. Blackwell Publishing Inc; 2011. p. 206.

134. Mohamed AR, Freeman JL, Maixner W, Bailey CA, Wrennall JA, Harvey AS. Temporoparietooccipital disconnection in children with intractable epilepsy: Clinical article. United States: American Association of Neurological Surgeons (1224 West Main Street Suite 450, Charlottesville VA 22903, United States); 2011. p. 660-70.

135. Morrison G, Duchowny M, Resnick T, Alvarez L, Jayakar P, Prats AR, et al. Epilepsy surgery in childhood. A report of 79 patients. SWITZERLAND: Division of Neurological Surgery, Miami Children's Hospital, Fla. 33155; 1992. p. 291-7.

136. Moseley BD, Nickels K, Wirrell E. Surgical outcomes for intractable epilepsy in children with infantile Spasms. John Wiley and Sons Inc; 2010. p. S126-S7.

137. Mukahira K, Oguni H, Awaya Y, Tanaka T, Saito K, Shimizu H, et al. Study on surgical treatment of intractable childhood epilepsy. Netherlands1998. p. 154-64.

138. Murakami H. Surgical treatment of refractory epilepsy in tuberous sclerosis complex. Japan2005. p. 229-39.

139. Nabangchang C, Saguankiat P, Sakoolnamarka S, Paueksakon P. Epilepsy surgery in children and adolescence: Pramongkutklao College of Medicine's experience. Elsevier; 2009. p. S108.

140. Nabangchang C, Sakoolnamarka S, Paueksakon P, Chinvarun Y. Epilepsy surgery in children and adolescence; Phramongkutklao College of Medicine's experience. Thailand2005. p. S263-70.

141. Nariai H, Nagasawa T, Juhasz C, Sood S, Chugani HT, Asano E. Statistical mapping of ictal high-frequency oscillations in epileptic spasms. United States: Blackwell Publishing Inc. (350 Main Street, Malden MA 02148, United States); 2011. p. 63-74.

142. Nilsson D, Fohlen M, Jalin C, Dorfmuller G, Bulteau C, Delalande O. Foramen ovale electrodes in the preoperative evaluation of temporal lobe epilepsy in children. United States: Blackwell Publishing Inc. (350 Main Street, Malden MA 02148, United States); 2009. p. 2085-96.

143. Oliveira RS, Santos MV, Terra VC, Sakamoto AC, Machado HR. Tailored resections for intractable rolandic cortex epilepsy in children: a single-center experience with 48 consecutive cases. Childs Nervous System. 2011;27(5):779-85.

144. Olsson I, Rydenhag B, Flink R. Seizure outcome after extratemporal lobe resections in Swedish children operated 1990 2004. W.B. Saunders Ltd; 2009. p. S3.

145. Otsuki T, Kaido T, Takahashi A, Kaneko Y, Nakagawa E, Sugai K, et al. Resective surgery for intractable epilepsy in children. Surgical prognosis. Blackwell Publishing Inc; 2009. p. 255.

146. Paolicchi JM, Jayakar P, Dean P, Yaylali I, Morrison G, Prats A, et al. Predictors of outcome in pediatric epilepsy surgery. United States2000. p. 642-7.

147. Park K, Buchhalter J, McClelland R, Raffel C. Frequency and significance of acute postoperative seizures following epilepsy surgery in children and adolescents. United States2002. p. 874-81.

148. Pasquier B, Peoch'h M, Fabre-Bocquentin B, Bensaadi L, Pasquier D, Hoffmann D, et al. Surgical pathology of drug-resistant partial epilepsy: A 10-year-experience with a series of 327 consecutive resections. France2002. p. 99-119.

149. Picot MC, Jaussent A, Fohlen M, Vall'e L, Villeneuve N, Motte J, et al. Two years outcomes of a multicenter cohort of non-surgical and post-surgical children with intractable epilepsy: Impact on family functioning and quality of life. John Libbey Eurotext; 2009. p. 171.

150. Piroddi C, Scarpa P, Pelle F, Cascardo B, Zanardi G, Francione S, et al. Cognitive variations in children after drug-resistant focal epilepsy surgery. Italy: Lega Italiana contro l'Epilessia (Via Di Rudini 8, Milan 20142, Italy); 2010. p. 50-6.

151. Pomata HB, Gonzalez R, Bartuluchi M, Petre CA, Ciraolo C, Caraballo R, et al. Extratemporal epilepsy in children: Candidate selection and surgical treatment. Germany2000. p. 842-50.

152. Prato G, Pinto F, Fazzini F, Piattelli G, Consales A, Rossi A, et al. The collaboration between neurologist and neuro-surgeon in child epilepsy. Italy: Lega Italiana contro l'Epilessia (Via Di Rudini 8, Milan 20142, Italy); 2008. p. 255-6.

153. Ramachandrannair R, Otsubo H, Shroff MM, Ochi A, Weiss SK, Rutka JT, et al. MEG predicts outcome following surgery for intractable epilepsy in with normal or nonfocal MRI findings. United States2007. p. 149-57.

154. Rasmussen T. Tailoring of cortical excisions for frontal lobe epilepsy. CANADA: Montreal Neurological Institute and Hospital, Quebec, Canada; 1991. p. 606-10.

155. Ribaric II, Nagulic M, Djurovic B. Surgical treatment of epilepsy: our experiences with 34 children. GERMANY: Neurosurgical Clinic, University Clinical Center, Belgrade, Yugoslavia; 1991. p. 402-4.

156. Rydenhag B, Hedstrm A, Olovson B, Uvebrant P, Viggedal G, Olsson I. Children with low IQ benefit from resective epilepsy surgery outcome at two-year follow-up in a consecutive series from Gteborg, Sweden, 1987-2004. Blackwell Publishing Inc; 2009. p. 46.

157. Sabaz M, Lawson JA, Cairns DR, Duchowny MS, Resnick TJ, Dean PM, et al. The impact of epilepsy surgery on quality of life in children. United States: School of Women's and Children's Health, University of New South Wales, Sydney, Australia; 2006. p. 557-61.

158. Sales LV, Velasco TR, Funayama S, Ribeiro LT, Andrade-Valenca LP, Neder L, et al. Relative frequency, clinical neuroimaging, and postsurgical features of pediatric temporal lobe epilepsy. Brazil2006. p. 1365-72.

159. Sinclair DB, Aronyk K, Snyder T, McKean J, Wheatley M, Bhargava R, et al. Pediatric temporal lobectomy for epilepsy. Switzerland2003. p. 195-205.

160. Sinclair DB, Aronyk KE, Snyder TJ, Wheatley BM, McKean JDS, Bhargava R, et al. Pediatric Epilepsy Surgery at the University of Alberta: 1988-2000. United States2003. p. 302-11.

161. Sinclair DB, Wheatley M, Snyder T, Gross D, Ahmed N. Posterior resection for childhood epilepsy. United States: Comprehensive Epilepsy Program, University of Alberta Hospital, Edmonton, Alberta, Canada; 2005. p. 257-63.

162. Skirrow C, Cross JH, Cormack F, Harkness W, Vargha-Khadem F, Baldeweg T. Long-term intellectual outcome after temporal lobe surgery in childhood. United States: Lippincott Williams and Wilkins (530 Walnut Street, Philadelphia PA 19106-3621, United States); 2011. p. 1330-7.

163. Smith ML, Elliott IM, Lach L. Cognitive, psychosocial, and family function one year after pediatric epilepsy surgery. United States2004. p. 650-60.

164. Smith ML, Kelly K, Kadis DS, Elliott IM, Olds J, Whiting S, et al. Self-reported symptoms of psychological well-being in young adults who underwent resective epilepsy surgery in childhood. United States: Blackwell Publishing Inc. (350 Main Street, Malden MA 02148, United States); 2011. p. 891-9.

165. Sondag M, Choi J, Koh S, Laux L, Alden T, Dipatri A, et al. Pediatric epilepsy surgical series of primarily ECoG-guided resections. John Libbey Eurotext; 2009. p. 171-2.

166. Sotero de MM, Connolly M, Bolanos A, Madsen J, Black PM, Riviello JJ, Jr. Temporal lobectomy in early childhood: The need for long-term follow-up. Canada2001. p. 585-90.

167. Stapleton SR, Kiriakopoulos E, Mikulis D, Drake JM, Hoffman HJ, Humphreys R, et al. Combined utility of functional MRI, cortical mapping, and frameless stereotaxy in the resection of lesions in eloquent areas of brain in children. Switzerland1997. p. 68-82.

168. Stefanova DP, Minkin K, Bojinova V, Bussarsky A, Penkov M, Penev L, et al. Epilepsy surgery in childhood is possible in a European country with limited resources. W.B. Saunders Ltd; 2011. p. S92.

169. Steinbok P, Gan PYC, Connolly MB, Carmant L, Barry SD, Rutka J, et al. Epilepsy surgery in the first 3 years of life: A Canadian survey. United States: Blackwell Publishing Inc. (350 Main Street, Malden MA 02148, United States); 2009. p. 1442-9.

170. Stepanova TS, Zemskaia AG, Grachev KV, Rogulov VA. Clinico-electrophysiologic analysis of the results of surgical treatment of focal epilepsy in children. Russian Federation1981. p. 25-32.

171. Sugimoto T, Otsubo H, Hwang PA, Hoffman HJ, Jay V, Snead IO. Outcome of epilepsy surgery in the first three years of life. United States1999. p. 560-5.

172. Szabo CA, Rothner AD, Kotagal P, Erenberg G, Dinner DS, Wyllie E. Symptomatic or cryptogenic partial epilepsy of childhood onset: fourteen-year follow-up. United States: Division of Neurology, University of Texas Health Science Center, San Antonio, Texas 78284-7883, USA; 2001. p. 264-9.

173. Szabo CA, Wyllie E, Stanford LD, Geckler C, Kotagal P, Comair YG, et al. Neuropsychological effect of temporal lobe resection in preadolescent children with epilepsy. United States1998. p. 814-9.

174. Takahashi A, Kaido K, Kaneko Y, Otsuki T, Nakagawa E, Sugai K, et al. Usefulness of SISCOM (subtraction ictal SPECT coregistered to MRI) in pediatric epilepsy surgery. Blackwell Publishing Inc; 2009. p. 449.

175. Ternier J, Pier KS, Boyd S, Cross H, Harkness W, Pressler R. A retrospective review of 86 cases of invasive recording at Great Ormond Street Hospital; An evaluation of complications and outcomes. Springer Verlag; 2009. p. 1358-9.

176. Terra V, Sakamoto A, Pinto K, Escorsi-Rosset S, Souza-Oliveira C, Wichert-Ana L, et al. Rolandic cortex and epilepsy: Resection limits. Springer Verlag; 2009. p. 1348.

177. Terra VC, Scorza FA, Cavalheiro EA, Wichert-Ana L, Pinto KGFD, MacHado HR, et al. Pediatric epilepsy surgery and sudden unexpected death epilepsy: The contribution of a Brazilian epilepsy surgery program. Germany: Springer Verlag (Tiergartenstrasse 17, Heidelberg D-69121, Germany); 2010. p. 1075-9.

178. Terra-Bustamante VC, Fernandes RMF, Inuzuka LM, Velasco TR, Alexandre Jr V, Wichert-Ana L, et al. Surgically amenable epilepsies in children and adolescents: Clinical, imaging, electrophysiological, and post-surgical outcome data. Germany2005. p. 546-51.

179. Terra-Bustamante VC, Inuzuca LM, Fernandes RMF, Funayama S, Escorsi-Rosset S, Wichert-Ana L, et al. Temporal lobe epilepsy surgery in children and adolescents: Clinical characteristics and post-surgical outcome. United Kingdom2005. p. 274-81.

180. Turon-Vinas E, Lopez-Casas A, Palacio-Navarro A, Donaire A, Garcia-Fructuoso G, Rumia J, et al. [Five years' experience in a paediatric epilepsy unit]. Experiencia de cinco anos en una unidad de epilepsia pediatrica. Spain: Servicio de Neurologia, Hospital Sant Joan de Deu, 08950 Esplugues de Llobregat, Espana. eturon@hsjdbcn.org; 2010. p. 451-60.

181. Uldall P. Influence of IQ/DQ on outcome in childhood epilepsy surgery. W.B. Saunders Ltd; 2009. p. S59.

182. Uldall PV, Nielsen H, Alving J, Buchholt JM, Herning GM, Hogenhaven H, et al. Epilepsy surgery of Danish children 1996-2002. Denmark2002. p. 5795-8.

183. van Empelen R, Jennekens-Schinkel A, Gorter JW, Volman MJM, van Nieuwenhuizen O, Helders PJM, et al. Epilepsy surgery does not harm motor performance of children and adolescents. England: Department of Paediatric Physical Therapy and Exercise Physiology, University Medical Centre, Wilhelmina Children's Hospital, Utrecht, The Netherlands. R.vanEmpelen@wkz.azu.nl; 2005. p. 1536-45.

184. van Empelen R, Jennekens-Schinkel A, van Rijen PC, Helders PJM, van Nieuwenhuizen O. Health-related quality of life and self-perceived competence of children assessed before and up to two years after epilepsy surgery. United States: Department of Pediatric Physical Therapy and Exercise Physiology, University Medical Center-Wilhelmina Children's Hospital, Utrecht, The Netherlands. R.vanEmpelen@wkz.azu.nl; 2005. p. 258-71.

185. Van Oijen M, De Waal H, Van Rijen PC, Jennekens-Schinkel A, van Huffelen AC, Van Nieuwenhuizen O, et al. Resective epilepsy surgery in childhood: the Dutch experience 1992-2002. England: Department of Child Neurology, Wilhelmina Children's Hospital, University Medical Center Utrecht, Utrecht, The Netherlands; 2006. p. 114-23.

186. Vossler DG, Wilkus RJ, Ojemann GA. Preoperative EEG correlates of seizure outcome from epilepsy surgery in children. United States1995. p. 236-45.

187. Whittle JR, Ellis HJ, Simpson DA. The surgical treatment of intractable childhood and adolescent epilepsy. Australia1981. p. 190-6.

188. Worrell GA, So EL, Kazemi J, O'Brien TJ, Mosewich RK, Cascino GD, et al. Focal ictal beta discharge on scalp EEG predicts excellent outcome of frontal lobe epilepsy surgery. United States: Section of Electroencephalography, Mayo Clinic, Rochester, Minnesota 55905, USA; 2002. p. 277-82.

189. Wyllie E, Luders H, Morris IH, Lesser RP, Dinner, D.S, et al. Subdural electrodes in the evaluation for epilepsy surgery in children and adults. Germany1988. p. 80-6.

190. Zaccariotti VA, Pannek HW, Holthausen H, Oppel F. Evaluation with subdural plates in children and adolescents. ENGLAND: Institutio de Neurologia de Goiania, Brazil; 1999. p. 463-74.

No seizure outcome data available (3)

1. Bein BN, Boreiko VB: The dynamics of temporal lobe epilepsy manifestations and the social-occupational prognosis in children following surgical removal of the epileptic focus*.* Russian Federation, 1975, pp 512-517.

2. Benifla M, Otsubo H, Ochi A, Weiss SK, Donner EJ, Shroff M, Chuang S, Hawkins C, Drake JM, Elliott I, Smith ML, Snead IO, Rutka JT: Temporal lobe surgery for intractable epilepsy in children: An analysis of outcomes in 126 children*.* United States, 2006, pp 1203-1213.

3. Cross JH, Colonnelli MC, D'Argenzio L, Hannan S, Harkness W: Psychiatric disorders in extra-temporal epilepsy, W.B. Saunders Ltd, 2009, p S76.

Anomalous feature in presented participants (1)

1. Carrazana EJ, Lombroso CT, Mikati M, Helmers S, Holmes GL, Genton P, Portera-Sanchez A: Facilitation of infantile spasms by partial seizures*.* United States, 1993, pp 97-109.

Participants did not undergo surgery (1)

1. Chen LS, Wang N, Lin M-I: Seizure outcome of intractable partial epilepsy in children*.* United States, Division of Neurology, Children's Hospital Los Angeles, Keck School of Medicine, University of Southern California, 90027, USA, 2002, pp 282-287.

Editorial (2)

1. Curatolo P: Intractable epilepsy in tuberous sclerosis: is the tuber removal not enough? England, Department of Neuroscience, Pediatric Neurology Unit, Tor Vergata University of Rome, Rome, Italy, 2010, p 987.

2. Mukawa J: Surgical treatment of epilepsy*.* JAPAN, Department of Neurosurgery, University of the Ryukyus School of Medicine, Okinawa, Japan, 1991, pp 201-211.

Abstract only (5)

1. D'Argenzio L, Colonnelli MC, Harkness W, Harrison S, Cross JH: Surgical treatment for paediatric extra-temporal epilepsy, Blackwell Publishing Inc, 2009, p 144.

2. Kessler S, Pieper T, Eitel H, Zsoter A, Zeches-Kansy C, Kudernatsch M, Kolodziejczyk D, Karlmeier A, Blmcke I, Holthausen H: 10 years pediatric epilepsy surgery: The Vogtareuth experience, Blackwell Publishing Inc, 2009, p 165.

3. Koskinen S, Putkonen P, Granstrm M, Gaily E: Quality of life after epilepsy surgery in children and adolescents, Blackwell Publishing Inc, 2009, p 142.

4. Kostiuk K, Tsymbaliuk V, Medvedev Y, Popov A, Zynkevich Y, Dichko S, Kanajkyn O: Choice of adequate surgical strategy in treatment of temporal lobe epilepsy, Blackwell Publishing Inc, 2011, pp 192-193.

5. Pieper T, Kessler S, Eitel H, Zsoter A, Zeches-Kansy C, Karlmeier A, Kudernatsch M, Kolodziejczyk D, Blmcke I, Holthausen H: 10 years pediatric epilepsy surgery- The Vogtareuth experience, Blackwell Publishing Inc, 2009, p 33.

Duplicate publication (5)

1. Huber Z: [Surgical treatment of symptomatic surgery in children using classical techniques versus extended lesion surgery and focal lesion surgery]: *Chirurgiczne leczenie padaczki objawowej u dzieci metoda klasycznych operacji czy tez poszerzona lezjonektomia wraz z fokektomia.* Poland, Oddzial Neurochirurgii Katedry Chirurgii Dzieciecej AM w Poznaniu, 2000, pp 75-84.

2. Li Y-L, Luan G-M, Zhou J, Bao M: [Pediatric intractable epilepsy surgery]*.* China, liyunlin6708@163.com, 2008, pp 210-212.

3. Liang S, Li A, Zhao M, Jiang H, Yu S, Meng X, et al. Epilepsy surgery in tuberous sclerosis complex: Emphasis on surgical candidate and neuropsychology. United States: Blackwell Publishing Inc. (350 Main Street, Malden MA 02148, United States); 2010. p. 2316-21.

4. Limbrick DD, Behdad A, Bertrand ME, Smyth MD: Epilepsy surgery in children with seizures arising from the rolandic cortex*.* United States, Blackwell Publishing Inc. (350 Main Street, Malden MA 02148, United States), 2009, pp 1450-1461.

5. van dH, van Huffelen AC, Spetgens WPJ, Ferrier CH, van Nieuwenhuizen O, Jansen FE: Identification of the epileptogenic zone in patients with tuberous sclerosis: concordance of interictal and ictal epileptiform activity*.* Netherlands, Department of Child Neurology, Rudolf Magnus Institute of Neuroscience, University Medical Centre, Utrecht, The Netherlands, 2010, pp 842-847.

Could not obtain IPD after contacting author (5)

1. Jahodova A, Krsek P, Marusic P, Tomasek M, Krijtova H, Kudr M, Petrak B, Rybar J, Tichy M, Komarek V. (2010) Surgical strategies in Tuberous Sclerosis patients: Effectiveness of extended tube-rectomias. Blackwell Publishing Inc, p. 31.

2. Jarrar RG, Buchhalter JR, Raffel C. (2004) Long-term outcome of epilepsy surgery in patients with tuberous sclerosis. United States, pp. 479-481.

3. Kyncl M, Krsek P, Jahodova A, Kudr M, Komarek V, Jayakar P, Dunoyer C, Resnick T, Altman N, Duchowny M. (2011) Magnetic resonance imaging localizes the epileptogenic zone in tuberous sclerosis complex. Blackwell Publishing Inc, p. 177.

4. Lee EH, Jeong M, Yum M, Ko T. (2009) Serial electroencephalographic findings of children with epilepsy associated with tuberous sclerosis. Blackwell Publishing Inc, p. 401.

5. Murakami H. (2005) Surgical treatment of refractory epilepsy in tuberous sclerosis complex. Japan, pp. 229-239.
