# Supplementary material for: Predictors of Seizure Outcomes in Children with Tuberous Sclerosis Complex and Intractable Epilepsy Undergoing Resective Epilepsy Surgery: An Individual Participant Data Meta-Analysis
Source: PLoS One. 2013 Feb 6;8(2):e53565. doi: 10.1371/journal.pone.0053565 (PMC3566144; doi:10.1371/journal.pone.0053565)
Supplement: Table S1 — Participant level data collection. (DOCX) [file pone.0053565.s004.docx]

**Table S2.**

| **Variable name** | **Type** | **Values and coding** | **Definition** |
| --- | --- | --- | --- |
| **Author** | Text | Text | Identifies the last name of the first author of the study |
| **Year** | Text | Text | Identifies the year of study publication |
| **Id** | Numeric (continuous) | Unique integer | Unique number for each record in each study |
| **Sex** | Numeric (binary) | 0 = female  1 = male | Gender of the study participant |
| **Ageszonset** | Numeric (continuous) | Integer  99 – missing data | Age of participant at seizure onset (months) |
| **Preopszfreq** | Numeric (continuous) | Integer  99 – missing data | Frequency of seizures preoperatively (per day) |
| **Is** | Numeric | 0 = no  1 = yes  8 = missing data | Identifies whether or not the child had infantile spasms |
| **Gsz** | Numeric | 0 = no  1 = yes  8 = missing data | Identifies whether or not the child had generalized seizure semiology |
| **Agesurgery** | Numeric (continuous) | Integer  99 – missing data | Age of participant at time of resective epilepsy surgery (years) |
| **Iq** | Numeric | Integer  0 = not performed  999 = missing data | Indicates the preoperative IQ score or the adaptive behavioral scale score (if IQ testing is not available) |
| **Ddlay** | Numeric | 0 = no or mild delay  1 = moderate/severe delay  8 = missing data | Degree of developmental delay as determined by cognitive tests or by level of schooling |
| **Surgerytype** | Numeric | 0 = lesionectomy/tuberectomy  1 = lobectomy  2 = multilobar resection  3 = hemispherectomy  8 = missing data | Uniquely identifies the type of operation performed |
| **InterictalEEG** | Numeric | 0 = normal/focal abnormality  1 = multifocal or generalized abnormality  8 = missing data | Indicates if the interictal EEG was normal, had a focal, multifocal or generalized abnormality |
| **IctalEEG** | Numeric | 0 = not performed  1 = focal  2 = multifocal or generalized  8 = missing data | Indicates if the ictal EEG had a focal, multifocal or generalized abnormality |
| **Tubersize** | Numeric (continuous) | 0 = MRI not performed  Integer  99 – missing data | Indicates the maximal size in mm of the predominant tuber on MRI if one is identified |
| **Tuberconcord** | Numeric | 0 = MRI not performed  1 = EEG/MRI non-concordant  2 = EEG/MRI concordant  999 = missing data | Indicates whether or not there is concordance between the MRI and EEG |
| **Tuberburden** | Numeric (continuous) | 0 = MRI not performed  Integer  99 = missing data | Identifies the number of tubers identified on MRI |
| **Fdgpet** | Numeric | 0 = not performed  1 = region of hypometabolism is not larger than the associated tuber  2 = region of hypometabolism is larger than the associated tuber  999 = missing data | Indicates whether or not the region of hypometabolism was larger than the associated tuber on FDG-PET study |
| **Spect** | Numeric | 0 = not performed  1 = unifocal abnormality  2 = multifocal abnormality  999 = missing data | Indicates whether the preoperative SPECT demonstrated a unifocal or multifocal abnormality |
| **Meg** | Numeric | 0 = not performed  1 = normal  2 = diffuse scatters  3 = unilobar cluster(s) at least one of which not associated with a tuber  4 = multilobar clusters at least one of which not associated with a tuber  5 = unilobar cluster(s) all of which are associated with a tuber  6 = multilobar cluster all of which are associated with tubers  999 = missing data | Indicates if the preoperative MEG was normal, demonstrated diffuse scatters or demonstrated cluster(s). If clusters were identified, it further indicates if they were uni- or multi- lobar, and whether or not they were associated with a tuber(s) |
| **Extraopinterictalecog** | Numeric | 0 = not performed  1 = normal/focal  2 = multifocal or non-focal  999 = missing data | Indicates if the interictal extraoperative ECOG was normal or had a focal, non-focal or multifocal abnormality |
| **Extraopictalecog** | Numeric | 0 = not performed  1 = no seizure captured/focal  2 = multifocal  999 = missing data | Indicates if the ictal extraoperative ECOG failed to capture a seizure or demonstrated a focal or multifocal electrographic seizure onset |
| **Durationfu** | Numeric (continuous) | Integer  99 = missing data | Duration of last follow-up after surgery (years) |
| **Outcome** | Numeric | 1 = Engel class I or II  2 = Engel class III or IV  8 = missing data | Uniquely categorizes the seizure outcome at the last clinical follow-up |
